# Comparative Transcriptome Analysis Reveals Cool Virulence Factors of *Ralstonia solanacearum* Race 3 Biovar 2

**DOI:** 10.1371/journal.pone.0139090

**Published:** 2015-10-07

**Authors:** Fanhong Meng, Lavanya Babujee, Jonathan M. Jacobs, Caitilyn Allen

**Affiliations:** Department of Plant Pathology, University of Wisconsin-Madison, Madison, Wisconsin, 53706, United States of America; Virginia Tech, UNITED STATES

## Abstract

While most strains of the plant pathogenic bacterium *Ralstonia solanacearum* are tropical, the race 3 biovar 2 (R3bv2) subgroup attacks plants in cooler climates. To identify mechanisms underlying this trait, we compared the transcriptional profiles of *R*. *solanacearum* R3bv2 strain UW551 and tropical strain GMI1000 at 20°C and 28°C, both in culture and during tomato pathogenesis. 4.2% of the ORFs in the UW551 genome and 7.9% of the GMI1000 ORFs were differentially expressed by temperature *in planta*. The two strains had distinct transcriptional responses to temperature change. GMI1000 up-regulated several stress response genes at 20°C, apparently struggling to cope with plant defenses. At the cooler temperature, R3bv2 strain UW551 up-regulated a cluster encoding a mannose-fucose binding lectin, LecM; a quorum sensing-dependent protein, AidA; and a related hypothetical protein, AidC. The last two genes are absent from the GMI1000 genome. In UW551, all three genes were positively regulated by the adjacent SolI/R quorum sensing system. These temperature-responsive genes were required for full virulence in R3bv2. Mutants lacking *lecM*, *aidA*, or *aidC* were each significantly more reduced in virulence on tomato at 20°C than at 28°C in both a naturalistic soil soak inoculation assay and when they were inoculated directly into tomato stems. The *lecM* and *aidC* mutants also survived poorly in potato tubers at the seed tuber storage temperature of 4°C, and the *lecM* mutant was defective in biofilm formation *in vitro*. Together, these results suggest novel mechanisms, including a lectin, are involved in the unique temperate epidemiology of R3bv2.

## Introduction

*Ralstonia solanacearum* is a soil-borne pathogen that causes bacterial wilt disease on more than 200 plant species, including important crops such as potato, tomato, eggplant, pepper, tobacco and banana [1]. The bacterium normally invades plant roots from the soil and spreads up into the stem and leaves through the host vascular system. It multiplies rapidly in the xylem vessels, reaching cell densities >10^9^ cfu g^−1^ of host tissue. Bacterial wilt is considered the single most destructive bacterial plant disease because of its aggressiveness, wide geographic distribution, and unusually broad host range [2]. Many virulence factors contribute to wilt disease development, including plant cell wall-degrading enzymes, bacterial extracellular polysaccharide (EPS), and a consortium of type III-secreted effectors [3]. These are regulated by a complex cascade that responds to unknown plant signal(s) and to cell density or confinement [4]. The role of temperature and other environmental signals in regulation of virulence factors has not been studied in *R*. *solanacearum*.

Most *R*. *solanacearum* strains cause disease in tropical to warm temperate environments, but a phenotypically and genetically homogenous subgroup of phylotype IIB, historically and for regulatory purposes known as Race 3 biovar 2 (R3bv2), is adapted to cooler environments and causes brown rot of potatoes in the highland tropics [5]. Brown rot is a major constraint to potato production in cool temperate climates worldwide, causing an estimated $950 million in losses each year [6]. R3bv2 can also infect tomato, geranium, and many weeds and wild plants [5]. Extensive field research documents that R3bv2 can cause disease at much cooler temperatures than tropical or subtropical *R*. *solanacearum* strains [7,8]. However, the mechanisms underlying this distinctive ecological trait are not understood.

R3bv2 and tropical strains have similar growth rates in culture at 20°C and 28°C and survive in comparable numbers in water at 4°C [9]. However, R3bv2 strain UW551 does survive longer in potato tubers at 4°C and is much more virulent on tomato at 20°C than tropical strains such as GMI1000 [9]. This suggests that the success of R3bv2 in temperate climates is mediated by interaction with host plants.

R3bv2 strains apparently originated in the cool Andean highlands [10,11]. R3bv2 has been accidentally introduced to North America and Europe via infected geranium cuttings imported from highland tropical areas, where the pathogen is endemic [12–15]. To date, R3bv2 is not established in North America. However, R3bv2 strains have survived for years in temperate European waterways in the weed host *Solanum dulcamara*, which sheds pathogen cells into surface water, triggering potato brown rot outbreaks [16–19]. Because of fears that R3bv2 may be able to overwinter and become established in American potato-growing regions, R3bv2 is listed as a U.S. Select Agent pathogen [20]. However, history suggests that quarantines often eventually fail [21–23]. Insights into the mechanisms of cool virulence in R3bv2 are needed to shape rational disease mitigation strategies.

*R*. *solanacearum* strains form a heterogenous species complex. Several strains have been sequenced, including GMI1000, a tropical strain belonging to the Asian phylotype I, sequevar 18 [24], and UW551, a typical cool virulent R3bv2 strain that belongs to American phylotype IIB, sequevar 1 [25]. Although both cause bacterial wilt disease of tomato, these two strains are quite evolutionarily divergent, with an average nucleotide identity around 91% [26]. Nonetheless, the synteny between the UW551 and GMI1000 genomes is 71%, and genes encoding most known bacterial wilt virulence determinants and regulators are highly conserved [25,27]. Comparison of the UW551 and GMI1000 genome sequences did not identify any differences in gene repertoire obviously linked to cold tolerance [25]. This suggested that the phenotypic differences in cold adaptation between R3bv2 and non-R3bv2 strains could be caused by genes of unknown function, or by differential regulation of orthologous genes [25].

To further explore this phenomenon, we used comparative transcriptome analysis to identify molecular mechanisms potentially involved in the cool virulence of R3bv2. We found that a small set of genes was differentially expressed by temperature in UW551 and GMI1000 during pathogenesis. There was little overlap between the temperature-responsive genesets in the two strains, suggesting that they have distinct responses to temperature. Mutational analysis revealed that several genes differentially expressed by temperature contributed significantly to the cool virulence of R3bv2, including a mannose-fucose binding lectin and two quorum sensing-regulated genes of unknown function.

## Results

To better understand the cool virulence mechanisms of *R*. *solanacearum* R3bv2, we profiled the transcriptomes of two biologically distinct strains of this pathogen, tropical strain GMI1000 and R3bv2 strain UW551, during pathogenesis of tomato plants at tropical (28°C) and temperate (20°C) temperatures. We also profiled the transcriptomes of both strains during growth in culture at the two temperatures. Because quorum sensing affects expression of several *R*. *solanacearum* virulence factors, we extracted RNA from bacteria at the same cell density under all conditions, ~6x10^8^ CFU g^−1^ or ml^−1^, when the bacterium’s quorum sensing systems should be active [28]. Four biological replicates of each strain were assayed for each of the four conditions, using whole genome microarray chips custom-designed for each strain. The experiments were highly reproducible, with average correlation coefficients across the *in planta* biological replicates of 0.978 and 0.933 for UW551 at 20°C and 28°C, and 0.96 and 0.948 for GMI1000 at 20°C and 28°C.

### Overview of genes differentially expressed (DE) by temperature

**Fig 1:**
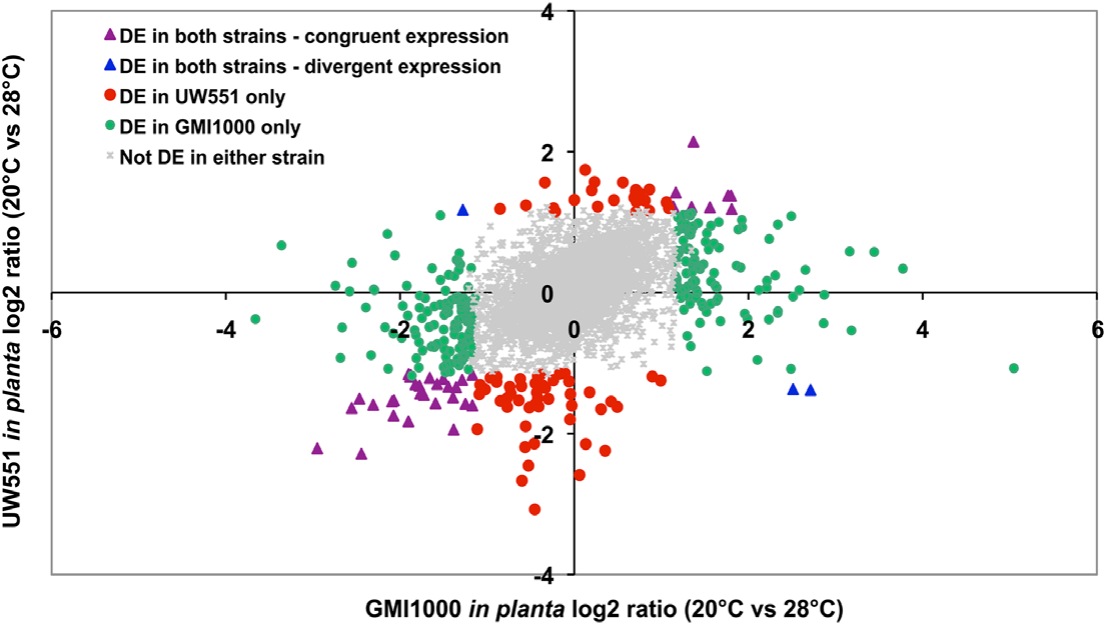
Scatter plot of log2 expression ratio for orthologous genes at 20°C compared to 28°C *in planta*. The orthologs shared between UW551 and GMI1000 were divided into five groups based on expression pattern. Gray dots in the middle represent orthologs not differentially expressed by temperature in either strain; symbols in colors represent orthologs differentially expressed in at least one strain. Each symbol represents one ortholog.

Temperature did not affect the expression of most genes in the two strains (Fig 1). Scatter plots showing mean signal intensities of all genes in UW551 and GMI1000 at 20°C and 28°C *in planta* had very high correlation coefficients (0.93 for UW551 and 0.91 for GMI1000) (S1 Fig). The in culture transcriptomes of both strains were also highly correlated at the two temperatures (data not shown). We used an EB-LNN critical threshold of 0.01 and a two-fold difference in expression to define differentially expressed (DE) genes. The differential expression of selected genes was confirmed with qPCR analysis of the same RNA samples used for the microarray analysis (S1 Table). *In planta*, tropical strain GMI1000 had more genes DE by temperature than temperate strain UW551. Overall, 4.2% of UW551 genes (181 of 4318) were DE by temperature *in planta* (S2 Table), and 3.2% (137 genes) were DE in culture (S3 Table). In strain GMI1000, 7.9% of genes (398 of 5061) were DE by temperature *in planta* (S4 Table), and 1.8% (89 genes) were DE in culture (S5 Table). There was little overlap between the GMI1000 and UW551 genesets that were DE by temperature *in planta* and in culture (S2 Fig), indicating that the tropical and temperate strains had distinct transcriptional responses to low temperature under the two experimental conditions. A COG analysis did not identify any noteworthy patterns, with the majority of DE genes in the ‘unassigned’ or ‘unknown function’ categories in all four conditions. Since responses in the host plant environment are most relevant to *R*. *solanacearum* biology, the following analysis focuses on the *in planta* data, unless otherwise noted.

Genomes of UW551 and GMI1000 share 3477 orthologous genes, and there are 816 strain-specific genes in UW551 and 1381 strain-specific genes in GMI1000 [29]. Most of the orthologs were not DE by temperature: only 3.3% (115 genes) in UW551 and 7.5% (263 genes) in GMI1000. There was minimal overlap between orthologs that were DE in both strains at low temperature, including 36 genes with congruent expression patterns and 3 genes with divergent expression patterns. These common DE genes included 24 from the *R*. *solanacearum* species complex Core Genome, as defined by analyses of 18 strains from all four phylotypes [27]. Genes encoding known bacterial wilt virulence factors like EPS, pectinases, and swimming and twitching motility were not DE by temperature in either strain, although several genes involved in Type 3 secretion were slightly upregulated at 20°C in both strains. Among the strain-specific genes, 66 (8.1%) were differentially expressed in UW551 and 135 (9.8%) were differentially expressed in GMI1000. In both strains, strain-specific genes were more often differentially expressed than orthologs in response to cool temperature, suggesting that UW551 and GMI1000 have fundamentally different responses to temperate conditions. This divergence could reflect distinct, independently evolved responses to chilling, and may offer useful clues to understanding the cool virulence trait of R3bv2 strains.

### Several stress and defense-related pathways were up-regulated at cool temperatures in tropical strain GMI1000

The *R*. *solanacearum nag* gene cluster, which putatively encodes degradation of the potent plant defense signal molecule salicylic acid, was also up-regulated in GMI1000 at 20°C (S6 Table). In addition, at the lower temperature GMI1000 had 3- to 6-fold higher expression of the genes encoding degradation of plant phenolic hydroxycinnamic acids (S3 Fig). Finally, several genes putatively or known to be involved in toxin efflux were more highly expressed in tropical strain GMI1000 during pathogenesis at 20°C than at 28°C (S7 Table). Collectively, this expression profile suggests that infecting a plant at cool temperatures is stressful for this tropical pathogen.

### R3bv2 genes induced by cool temperature

**Fig 2:**
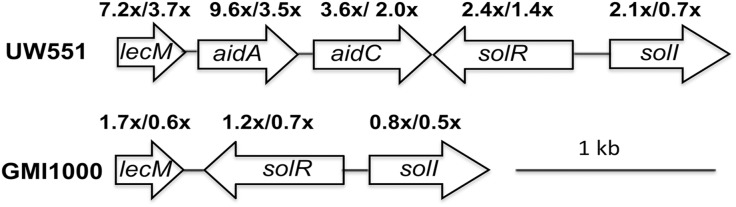
A cluster of genes adjacent to those encoding the SolI/R quorum sensing system were up-regulated in *R*. *solanacearum* strain UW551 at 20°C, in culture. Some were also upregulated *in planta*. Arrows represent open reading frames. The numbers above the arrows indicate the expression fold-change for each gene at 20°C compared to 28°C, in culture/*in planta*, determined by whole-genome microarray analysis as described in the text. The arrangement and expression levels of the corresponding genes in strain GMI1000 are also shown.

Genes encoding the SolI/R quorum sensing system were differentially up-regulated at 20°C relative to 28°C in temperate strain UW551 in culture, indicating that their expression is responsive to low temperature (Fig 2). An adjacent gene cluster, which was upregulated at 20°C both in culture and *in planta*, encodes LecM, a mannose-fucose binding lectin, and AidA and AidC, two proteins of unknown function. The GMI1000 genome does not contain either *aidA* or *aidC*, and GMI1000’s *lecM* homolog was not differentially expressed by temperature. We confirmed the microarray expression results for *lecM*, *aidA*, and *aidC* by measuring their expression in culture at both temperatures using quantitative real-time PCR (qPCR). For each gene, the expression trend was consistent between microarray and qPCR, although qPCR indicated a higher fold-induction (S1 Table), probably due to the higher sensitivity of the method. These results suggested that this region could be involved in cool virulence and thus warranted further study.

### Mutation of *lecM*, *aidA*, or *aidC* differentially reduced UW551 virulence at 20°C

**Fig 3:**
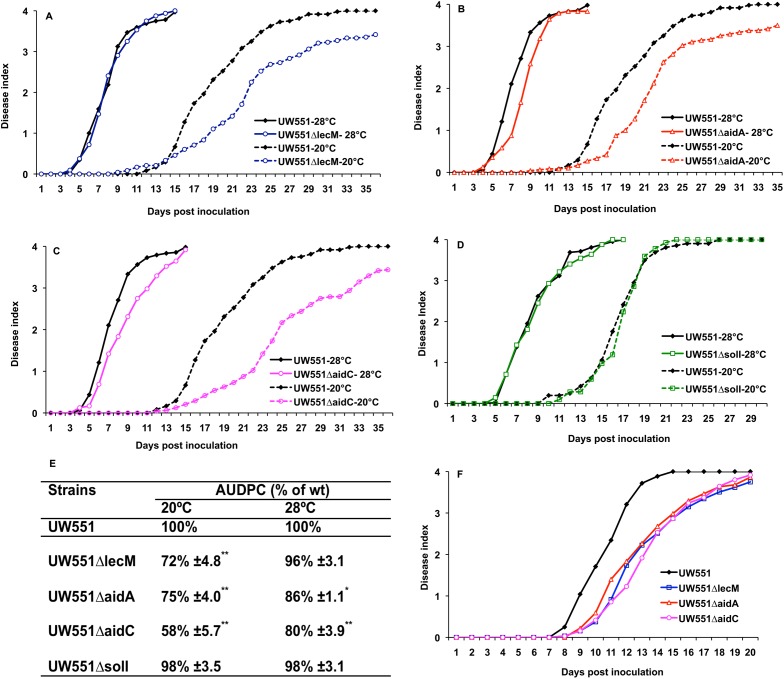
Mutation of *R*. *solanacearum* strain UW551 *lecM*, *aidA*, or *aidC* but not *solI* differentially reduced bacterial virulence at 20°C. Virulence was measured on wilt-susceptible tomato plants at 20°C and 28°C via soil soak inoculation (A-E) or at 20°C via cut petiole inoculation (F). Each point represents the mean of three biological replicates, each containing 16 plants per strain per temperature. The area under disease progress curve (AUDPC) was measured for each strain in A-C and each mutant’s AUDPC relative to wild type is shown (E). Asterisks indicate that virulence of wild type and mutant strains were significantly different (* *P*< 0.05, ** *P*< 0.01, ANOVA). Each mutant was also significantly reduced in virulence (*P*< 0.01, repeated measures ANOVA) compared to the wild type following cut petiole inoculation (F).

To test the hypothesis that genes in the lectin region contribute to the cool virulence of UW551, we generated deletion mutant strains UW551ΔlecM, UW551ΔaidA, and UW551ΔaidC, as well as a UW551ΔsolI mutant. qPCR analysis of cDNA from the mutants confirmed that there was no detectable expression of the deleted gene in each mutant. All the mutants grew indistinguishably from wild type in rich broth culture and in tomato leaves at both 20°C and 28°C, indicating that their phenotypes were not the result of a growth defect in culture or *in planta*. We measured the virulence of mutants on tomato plants using a biologically representative soil-soak inoculation that requires bacteria to actively find and invade unwounded host plant roots from the soil. At 28°C, UW551ΔlecM was almost as virulent as its wild type parent, while UW551ΔaidA and UW551ΔaidC had slight virulence defects. In contrast, at 20°C, all three mutants were significantly reduced in virulence relative to wild type (Fig 3). We quantified this differential virulence by comparing the area under the disease progress curve (AUDPC) for each strain at both temperatures to that of wild type. Strains lacking *lecM*, *aidA*, or *aidC* caused significantly less disease under temperate conditions (*P*<0.01). For example, at 28°C the AUDPC of UW551ΔlecM was 96% of wild type, but it was only 72% of wild type at 20°C (Fig 3E). All three mutants were also significantly reduced in virulence when they were inoculated directly into the tomato xylem via a cut leaf petiole (Fig 3F, *P*< 0.01, repeated measures ANOVA), indicating that R3bv2 strain UW551 requires *lecM*, *aidA*, and *aidC* for mid-stage bacterial wilt disease virulence, and not only for early infection. Together, these results demonstrate that *lecM*, *aidA*, and *aidC* encode functions necessary for cool virulence of UW551.

### Lectin-specific sugars inhibited *R*. *solanacearum* attachment to tomato roots

Lectins, which are carbohydrate-binding proteins, play important roles in cell–cell interactions, including microbial pathogenesis [30]. To test the hypothesis that LecM contributes to *R*. *solanacearum* adhesion to plants, we compared the ability of UW551 and the UW551ΔlecM mutant to attach to tomato seedling roots at 20°C, both separately and in a 1:1 competition assay. The *lecM*-deficient strain had no measurable defect in attachment in either condition. This could be the result of functional redundancy between LecM and LecX, the other mannose-fucose binding lectin encoded by the UW551 genome, especially since *lecX* was also strongly up-regulated *in planta* compared to its expression in CPG culture at the same temperature (Table 1).

**Table 1:** Expression of lectin genes in *R*. *solanacearum* strains GMI1000 and UW551 under various conditions.

Gene name	GMI1000 gene expression				UW551 gene expression				Locus tag	
	20°C CPG	28°C CPG	20°C IP	28°C IP	20°C CPG	28°C CPG	20°C IP	28°C IP	GMI1000	UW551
*lecF*	16.7^a^	16.6	16.7	16.6	*np* ^b^	*np*	*np*	*np*	RSc2107	*np*
*lecM*	14.5	13.8	15.9	16.5	13.3	10.5	14.6	12.6	RSc3288	RRSL_02788
*lecX*	16.7	16.6	16.8	16.6	14.9	14.7	16.2	16.1	RS03909	RRSL_03943

^a^Values shown are scaled log2 signal intensities from strain-specific whole genome microarrays hybridized to labeled cDNA extracted from *R*. *solanacearum* cells grown in rich culture medium (CPG) or *in planta* (IP). These absolute expression values were used to allow comparisons across strains.

^b^ *np*: gene not present in this strain.

LecM is 70% identical to LecB of the opportunistic human pathogen *Pseudomonas aeruginosa*; LecB contributes to *P*. *aeruginosa* virulence via involvement in biofilm formation, adhesion, host recognition, and inhibition of host defenses [31] [32–34]. Patients infected by *P*. *aeruginosa* have been successfully treated with a solution of sugars specifically bound by its two lectins, LecA and LecB [35,36]. The sugars compete for and block the lectin sugar-binding sites, thus inhibiting bacterial attachment to host cells.

To determine if lectins have a similar function in *R*. *solanacearum*, we incubated strain UW551 in a solution of sugars that bind to its two lectins (LecM and LecX) to see if this would affect its ability to attach to plant roots. UW551 cells pre-incubated with 10 mM D-mannose and 10 mM L-fucose were significantly reduced in attachment to tomato roots compared with the untreated bacteria (1.5x10^7^ cfu/g root as compared to 2.2x10^6^ cfu/g root, P<0.01, t-test). Exposure to the sugar solution did not affect bacterial viability and a control treatment with 10 mM glucose had no effect on bacterial attachment.

### LecM was required for biofilm formation *in vitro*

*R*. *solanacearum* forms biofilms on PVC plastic plates and biofilm-like aggregations on tomato seedling roots, although the biological significance of biofilms in this species is not known [37,38]. *P*. *aeruginosa* LecB specifically binds to cells in biofilms and contributes to biofilm formation. Exopolysaccharides are key components of microbial biofilms, and mannose, which is preferentially bound by both LecB and LecM, is a primary constituent of EPS from both *P*. *aeruginosa* and *R*. *solanacearum* [39–41]. We therefore tested the hypothesis that LecM is involved in biofilm formation. On PVC microtiter plates, UW551ΔlecM formed 9.15-fold less biofilm than its wild type parent (*P*<0.01). Together, these results suggest that LecM may function both in attaching bacteria to host plant cells (adhesion), and in bacterial cell–cell binding in biofilms (cohesion).

### LecM was required for normal host colonization

To measure the effect of LecM on bacterial success inside host plants, after the adhesion and entry stages, we quantified *R*. *solanacearum* population growth in stems of tomato inoculated directly into the xylem via a cut petiole with either UW551 or UW551ΔlecM and incubated at 20°C. Two days after inoculation, plants inoculated with wild type and UW551ΔlecM contained similar numbers of bacteria, with mean population sizes just over 1×10^6^ cfu g^−1^. However, eight days after inoculation, the mean population size of UW551 in tomato stem was 2.83×10^11^ cfu g^−1^, more than 50-fold larger than that of UW551ΔlecM, which was 5x10^9^ cfu g^−1^ (*P*<0.01). This result indicated that *lecM* contributes to xylem colonization by UW551. The colonization defect at day 8 corresponds to the difference in time of disease onset between wild type and UW551ΔlecM in the cut-petiole virulence assay (Fig 3F).

### UW551ΔlecM and UW551ΔaidC survived poorly in potato tubers at 4°C

**Fig 4:**
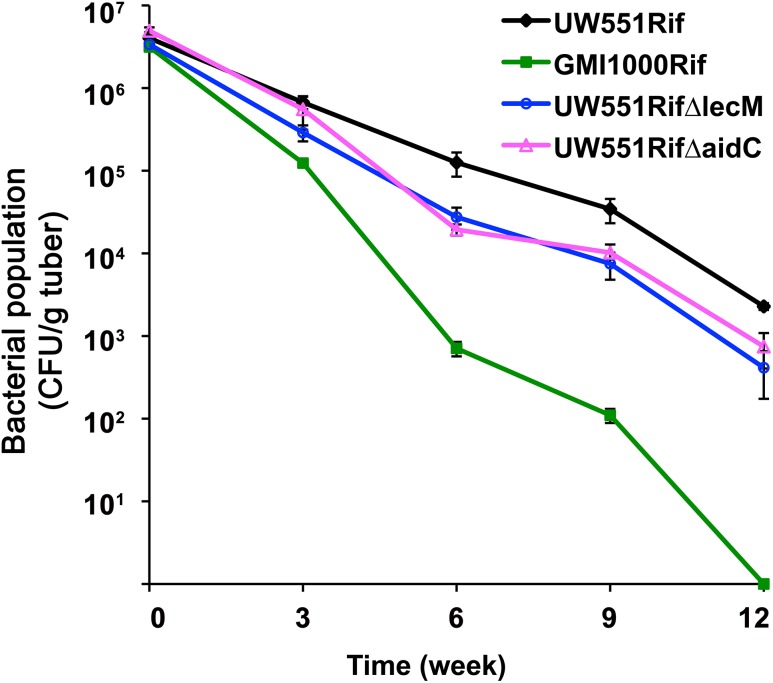
UW551ΔlecM and UW551ΔaidC had reduced survival in potato tubers at 4°C. Potato tubers were injected with different *R*. *solanacearum* strains, and bacterial cell numbers were counted by grinding and dilution plating tubers at different times after inoculation. The experiment was repeated three times, with three tubers per strain per time point. At 6, 9 and 12 weeks after inoculation, the population sizes of UW551ΔlecM and UW551ΔaidC in tubers were significantly lower than those of the wild type parent strain (*P*<0.05, ANOVA).

We previously found that R3bv2 strain UW551 survived much longer than non-R3bv2 strains in tubers of potato, a socioeconomically important natural host, at the common seed tuber storage temperature of 4°C [9]. We inoculated potato tubers with *lecM* and *aidC* mutants and measured their population sizes over time. As previously observed, tropical strain GMI1000 survived poorly in potato tubers at 4°C compared to UW551. At 6, 9 and 12 weeks after inoculation, the population sizes of UW551ΔlecM and UW551ΔaidC in tubers were around 5-fold lower than those of the wild type parent strain (Fig 4, *P*<0.05), indicating that LecM and AidC contribute to cold survival of UW551 as well as to temperate virulence.

### Adding the UW551 lectin region to GMI1000 did not confer cool virulence on GMI1000

Since *aidA* and *aidC* are not present in GMI1000 and there is some sequence variation between the two strains in this region, we tested the hypothesis that inserting the UW551 *lecM* cluster into a neutral site in the GMI1000 chromosome would increase its virulence in temperate conditions. However, neither a fragment containing the UW551 *lecM*, *aidA*, and *aidC* ORFs (plus 753 bp of upstream sequence) nor a larger fragment containing the UW551 *lecM*, *aidA*, *aidC*, *soIR*, and *solI* ORFs increased the virulence of GMI1000 at 20°C. This negative result suggests that either the necessary UW551-typical regulation of these genes did not occur in GMI1000, or that additional elements are required to confer cool virulence on non-R3bv2 strains.

### LecM, AidA, and AidC are required for cool virulence in other R3bv2 strains

We attempted to complement each of these three mutations, first *in trans* on a low-copy number plasmid and then by insertion of single copies into the selectively neutral chromosomal *att* site. In all cases the merodiploid strains had pleiotropic phenotypes: they were hypermotile and several overproduced an acylhomoserine lactone quorum sensing signal molecule. We therefore used an alternative method to confirm that the insertions in the target genes were responsible for the cool virulence phenotypes we observed, and to demonstrate that this result was not unique to UW551. We independently recreated the *lecM*, *aidA*, and *aidC* mutations in two additional R3bv2 strains of *R*. *solanacearum*, UW553 and UW560. The resulting mutants did not have the pleiotropic phenotypes observed in the merodiploid “complemented” strains, nor did they have any growth defect compared to the wild type strains. We tested the virulence of these mutants at 20°C via soil-soak inoculation. Consistent with the findings in UW551, strain UW553 and UW560 mutants lacking *lecM*, *aidA*, or *aidC* had similarly reduced virulence at 20°C compared to their respective wild type parents (S4 Fig, *P*< 0.01, repeated measures ANOVA). These results confirmed that each of these genes is important for the temperate virulence of R3bv2 strains.

### The l*ecM* region is regulated by the SolI/R quorum sensing system

Since the *lecM* gene cluster is adjacent to the *solI/R* acyl-homoserine lactone (AHL) quorum sensing (QS) system, and *aidA* is controlled by *solI/R* [28], we used qRT-PCR to determine if other genes in this region were also regulated by SolI/R. When *solI* was deleted, transcription in culture of *lecM*, *aidA*, and *aidC* were all lower than in a wild-type background, although to differing degrees. Expression of *lecM* was 54.7-fold lower in UW551ΔsolI, while *aidA* expression was 31-fold lower and *aidC* was 2.2-fold lower. These results demonstrate that, at least in culture, *lecM*, *aidA*, and *aidC* are all positively regulated by the SolI/R QS system.

## Discussion

We compared the transcriptional profiles of two ecologically distinct strains of a high-impact plant pathogen at different temperatures in culture and during disease in a natural host. There was only minimal overlap between the differentially expressed gene sets in culture and *in planta* for both temperate R3bv2 strain UW551 and tropical strain GMI1000, confirming our previous finding that *R*. *solanacearum* regulates gene expression very differently *in vitro* and *in planta* [29]. These results offer further evidence that data from *in vitro* studies do not reliably reflect pathogen behavior *in vivo* and therefore should be used and interpreted with caution [42].

This *in planta* comparative analysis yielded insights into ways that temperature has shaped two related pathogens. Changing the temperature of pathogenesis from a tropical 28°C to a cool temperate 20°C altered expression of relatively few genes in both *R*. *solanacearum* strains. Nonetheless, almost twice as many genes were differentially expressed at the lower temperature in GMI1000 than in UW551, and the number of genes up-regulated at 20°C in GMI1000 was almost three times greater than the number up-regulated in UW551. This was not unexpected, since UW551 is a successful pathogen at both temperate and tropical temperatures, and is thus well adapted to broad environmental parameters. When UW551 causes disease at 20°C, it alters expression of less than 5% of its genome relative to when it wilts tomato plants at 28°C. In contrast, tropical strain GMI1000 had a more significant transcriptional response to cool temperatures, including up-regulating diverse stress-related functions, possibly because it is not adapted to this condition.

The transcriptional response of prokaryotes to subtle yet physiologically relevant temperature changes is largely unexplored even for well-studied model organisms. Essentially nothing is known about traits required by phytopathogenic bacteria for successful pathogenesis at lower temperatures. Studies of bacterial adaptation to extreme low temperatures typically identify genes encoding cold shock proteins, membrane lipid desaturases, and molecular chaperones that stabilize proteins at near-freezing temperatures [43–46]. However, none of these genes were differentially expressed by temperature in either *R*. *solanacearum* strain, possibly because our experiment did not expose bacteria to a sudden change in temperature. It is also likely that 20°C was not low enough to induce cold shock.

*R*. *solanacearum* strain GMI1000 is only weakly virulent on tomato at 20°C [9]. At this cool temperature, the tropical strain increased expression of diverse self-protective functions that were not up-regulated in temperate strain UW551 under the same condition. Like most bacteria, *R*. *solanacearum* strains have multiple toxin efflux pumps that confer tolerance of deleterious compounds and are often up-regulated as part of a generalized stress response [47,48]. We previously found that AcrAB, an RND family efflux pump, contributes to bacterial wilt virulence by protecting *R*. *solanacearum* from host antimicrobial compounds [49]. Genes encoding six toxin efflux pumps, including AcrAB, were differentially up-regulated during GMI1000 pathogenesis of tomato at 20°C, suggesting that this tropical *R*. *solanacearum* strain confronts toxic plant defenses in temperate conditions.

The *nag* genes, which encode degradation of the plant signaling molecule salicylic acid (SA) were also differentially up-regulated at 20°C in GMI1000. The SA defense pathway is activated during tomato resistance to *R*. *solanacearum* [50], and SA itself can directly reduce bacterial fitness and virulence factor production [51]. Elevated *nag* cluster expression may reflect a bacterial response to high SA levels in tomato plants infected by GMI1000 at 20°C. We previously found that at 28°C, GMI1000-infected tomato plants expressed SA defense genes faster and to a greater degree than plants infected with UW551 [52]. At 28°C, both GMI1000 and UW551 can successfully overcome SA-dependent defenses in tomato, and UW551 is still able to do this at 20°C. GMI1000 may increase expression of its SA degradation pathway at 20°C in an unsuccessful attempt to overcome SA-triggered plant defenses.

Finally, during pathogenesis at 20°C, GMI1000 differentially upregulated genes encoding degradation of hydroxycinnamic acids, which are major plant cell wall-reinforcing phenolic compounds that also have direct antimicrobial properties [53–58]. *R*. *solanacearum* elicits production of cell-wall-bound phenolic compounds in tomato, and increased phenolic levels correlated with lower *R*. *solanacearum* population sizes [59,60]. Furthermore, we recently found that the ability to degrade hydroxycinnamic acids is required for full virulence of GMI1000 at 28°C [61]. The increased expression of the hydroxycinnamic acid degradation pathway at 20°C suggests that GMI1000 may confront higher levels of phenolics at cool temperatures, where it struggles to cause disease. Consistent with this idea, we observed that tomato plants infected by GMI1000 at 20°C exhibit extensive vascular browning, which is indicative of plant phenolic production. We speculate that, like resistant plants, tomatoes infected with GMI1000 at 20°C produce more phenolic compounds. This may help explain the strain’s weak virulence at this temperature.

Taken together, this expression profile suggests that GMI1000 confronts significant stress in tomato plants at 20°C, and it may up-regulate toxin efflux pumps and enzymes that degrade SA and phenolic compounds in an ineffectual attempt to overcome host defenses. Alternatively, GMI1000 and UW551 may experience similar levels of host defense under these conditions, but UW551 is better able to tolerate them.

A recent study that compared the proteomes of tropical and cool virulent *R*. *solanacearum* strains at 18°C and 30°C identified 101 proteins that were differentially expressed by temperature [62]. This proteomic analysis found that Type 3 secretion system structural protein HrcC is more abundant in cool virulent strains at the lower temperature, in agreement with our findings. However, the list of differentially expressed proteins did not generally correspond to the genes identified by our transcriptional analysis. This may reflect the slightly different temperatures used in the two studies (20/28°C vs. 18/30°C) or the differences between levels of protein and mRNA. It is more likely that the contrasting results of these two studies can be explained by their very different experimental designs. The proteomic analysis quantified proteins extracted from bacteria co-cultured with tomato seedlings in static liquid MS medium, whereas the transcriptomic study measured mRNA from bacteria extracted from the xylem vessels of diseased whole tomato plants. Thus, both the *R*. *solanacearum* strains and their host plants experienced significantly different physiological conditions in these two studies.

A separate study found that cool virulent strains exhibited reduced twitching motility, which is required for bacterial wilt virulence [63]. However, no known twitching motility genes were DE by temperature in either the cool virulent R3bv2 strain or tropical strain GMI1000. Again, the experimental conditions were not comparable; cool virulent strains may use twitching motility on solid surfaces like agar plates (or plant roots) but not in shaking broth culture or in tomato xylem, which were the two conditions we assayed here.

Three genes in a cluster that was up-regulated at 20°C in R3bv2 strain UW551 contributed significantly to cool virulence on tomato plants and to survival in potato tubers at 4°C. To our knowledge *lecM*, *aidA*, and *aidC* are the first genes known to be involved in temperature adaptation in plant pathogenic bacteria. However, their exact mechanisms of action remain to be determined. Our data suggest that the mannose/fucose binding lectin LecM increases pathogen attachment to other bacteria during biofilm formation. Pre-treatment of the bacterium with a solution of mannose and fucose reduced root attachment, which is indirect evidence that that lectins may also help *R*. *solanacearum* attach to host surfaces.

Binding affinities and crystal structures have been determined for three lectins from *R*. *solanacearum*, originally named RSL, RS-IIL, and RS20L, but their biological functions remain unknown. LecF (RSL), which is not present in UW551, preferably binds L-fucose [64–66]. LecX (RS20L) binds L-fucose, D-mannose and D-xylose [67]. LecM (RS-IIL), which was significantly induced in UW551 at 20°C, binds D-mannose, L-fructose, L-fucose and D-arabinose [65]. These sugars are common constituents of plant cell wall polysaccharides [68,69].

Interestingly, all lectin genes from both strains were highly expressed under all conditions tested, although with differing expression patterns. Specifically, *lecF* and *lecX* were consistently highly expressed in GMI1000; indeed, their expression levels were so high that the chip may have been saturated, preventing detection of expression differences across conditions. In UW551, *lecX* was up-regulated *in planta*, but was not differentially expressed by temperature, and *lecM* was up-regulated in response to both growth *in planta* and low temperature. *lecM* was also induced *in planta* in GMI1000, though not by low temperature. The high expression levels of all lectin genes suggest these proteins are important for *R*. *solanacearum* fitness *in planta*. It would be interesting to determine the virulence of a triple mutant lacking all three lectin structural genes.

Plant lectins play important diverse roles in plant-microbe interactions [70–73], but to our knowledge this is the first indication that bacterial lectins may contribute to pathogen attachment to plants. Our finding that lectin-specific sugars could inhibit attachment of UW551 cells to tomato roots is consistent with a novel role for *R*. *solanacearum* lectins in bacterial adherence to plant cells. Because the UW551ΔlecM mutant was significantly reduced in virulence even when it was introduced directly into the tomato xylem through a cut petiole, this lectin apparently also functions inside the plant, not only during initial root attachment.

The regulation of *R*. *solanacearum lecM* may resemble that of its *P*. *aeruginosa* homolog, *lecB*. Like *lecM*, *lecB* is controlled by quorum sensing, but *lecB* is additionally regulated by the alternative sigma factor RpoS [74]. Expression of *R*. *solanacearum* SolI/R depends on RpoS [75], so *lecM* may also be indirectly regulated by RpoS via quorum sensing. In addition, an *in vitro* microarray study found that in GMI1000 *lecM* is regulated by the global virulence transcriptional regulator HrpG [76]. The fact that *lecM* expression is controlled by several regulators suggests the importance of this protein to bacterial fitness.

AidA and AidC were previously wholly cryptic, but this work demonstrated that they both play significant roles in bacterial wilt virulence, contributing disproportionately at low temperatures. They are not, however, unique to cool-temperate R3bv2 strains of *R*. *solanacearum*. Although *aidA* and *aidC* are absent from African (phylotype III) and Asian (phylotype I) strains, they are present in tropical Indonesian phylotype IV strains and in tropical and warm-temperate phylotype II strains from North and South America, suggesting that their function extends beyond cool virulence [26]. The *lecM-aidA-aidC* gene order is retained in *Burkholderia cenocepacia* and *Pseudomonas entomophila*, although the gene sequences are not well conserved. The *aidA* gene was originally identified in *R*. *solanacearum* strain AW1 because it is regulated by the SolIR quorum sensing system [28]. An AidA homolog in *Burkholderia cenocepacia* contributes to slow killing of nematodes [77]. However, *R*. *solanacearum* AidA is not necessary for slow killing of nematodes and its function remains unknown [78]. AidC is about 24% identical to AidA at the amino acid level and its function is also unknown; *aidC* is located directly downstream of *aidA* in all *R*. *solanacearum* genomes that contain *aidA*.

The SolI/R quorum sensing system regulated expression of *lecM*, *aidA*, and *aidC* at 20°C in strain UW551. Consistent with a previous study of a *solI* mutant in *R*. *solanacearum* strain AW1 [28], we found that the UW551Δ*solI* mutant had no virulence defect at either 20°C or 28°C. This might seem counterintuitive, since SolI/R positively regulates expression of *lecM*, *aidA*, and *aidC*, which were all required for virulence, especially at 20°C. Our microarrays and qPCR assays measured the transcription of *R*. *solanacearum* cells at densities higher than 1x10^8^ cfu g^−1^, which is above the threshold for activation of the SolI/R regulatory system. However, expression of these genes was induced not only by quorum sensing but also by low temperature and by a condition or signal present in plants, so it is possible that *lecM*, *aidA*, and *aidC* are expressed *in planta* even in the absence of *solIR*. We and others previously showed that virulence traits controlled by another quorum sensing regulator, PhcA, are regulated very differently in culture and *in planta* [29,79]. It is also possible that inactivation of *solIR* did not affect virulence because of redundancy between the virulence-regulating quorum sensing systems in *R*. *solanacearum*. The AHL-responsive SolI/R autoinduction system in *R*. *solanacearum* is part of a more complex autoregulatory hierarchy. Expression of *solR* and *solI* requires PhcA, which is itself controlled by a second quorum sensing system that responds to 3-OH-PAME [28]. As mentioned above, *solIR* expression is additionally dependent on the alternate sigma factor RpoS [75].

Overall, these studies reveal that cool virulence is a complex multigenic trait in *R*. *solanacearum*. Unexpectedly, we found that a bacterial lectin contributes differentially to bacterial wilt virulence at lower temperatures. Our results generated diverse testable hypotheses for further laboratory and field studies and also identified biochemical pathways that plant breeders may be able to manipulate in tomato varieties to restrict growth of vascular pathogens and generate disease-resistant crops.

## Materials and Methods

### Bacterial strains and culture conditions

The bacterial strains used in this study are listed in S8 Table. *R*. *solanacearum* strains were grown at 28°C either in casamino acids-peptone-glucose (CPG) rich broth or on tetrazolium chloride (TZC) plates, as previously described [80]. *Escherichia coli* strains were grown in Luria-Bertani medium [81] at 37°C. Antibiotics were added when needed at the following concentrations: ampicillin, 50 mg l^−1^; kanamycin, 25 mg l^−1^; tetracycline, 12.5 mg l^−1^, and rifampin, 25 mg l^−1^. Unless otherwise noted, medium components were purchased from Difco Laboratories (Detroit, MI) and other chemicals were purchased from Sigma-Aldrich (St. Louis, MO).

### RNA extraction

For the in culture samples, RNA was extracted from 30 ml cultures of strains UW551 and GMI1000 grown in rich CPG broth to log phase (~6x10^8^ cfu ml^−1^), using a modified hot-phenol extraction followed by four DNase treatments [82]. For the *in planta* samples, wilt-susceptible Bonny Best tomatoes were soil-soak inoculated at 28°C and 20°C with strain UW551 or strain GMI1000 as described for the virulence assay below. Before inoculation, roots of plants inoculated with GMI1000 at 20°C were disturbed by gently lifting the plant stem a few mm to increase numbers of symptomatic plants at this non-conducive temperature. Bacterial RNA was harvested from plants showing early wilting symptoms (disease index = 1, as described below). Since xylem vessels are the primary habitat of *R*. *solanacearum* during pathogenesis, we harvested bacteria from infected plants by centrifuging 7 cm sections of infected tomato stems at 13,500 *g* for 5 min in 15 ml conical tubes containing 3 ml transcriptional stop solution (95% ethanol, 5% phenol). The resulting bacterial pellet was stored at -80°C until the bacterial population size in the stem was confirmed by dilution plating a ground stem section. Pellets from stems colonized with 1x10^8^ cfu g^−1^ to 1x10^9^ cfu g^−1^ were pooled for RNA extraction by the hot phenol method. Absence of genomic DNA contamination in RNA was confirmed by PCR with *R*. *solanacearum* universal primers 759/760 [10]. Quality of RNA samples was assessed using Nanodrop and the Agilent Bioanalyzer 2100 nanochip system (Agilent Technologies). RNA samples with A260/A280 >2.0, A260/A230 >2.0, and RNA Integrity Number (RIN) >8 were used for microarray and qPCR analysis. We did four biological replicates for each condition of the microarray experiment.

### Transcriptome analysis with microarrays

The custom-designed DNA microarrays used for transcriptional analysis of strains GMI1000 and UW551 were previously described [29]. Synthesis of cDNA, labeling, hybridization, and scanning were conducted as previously described [29]. NimbleScan 2.4 software was used to extract the raw data from the scanned images and compute expression values (gene calls) according to the Robust Multichip Average (RMA) algorithm [83]. Signals from the four biological replicates of each experiment were normalized using quantile normalization [84] with the RMA procedure in NimbleScan 2.4. To identify genes differentially expressed between 28°C and 20°C in each strain, an empirical Bayes analysis, EBArrays [85], was executed within the R statistical analysis software package and Bioconductor v2.1 [86]. In culture and *in planta* growth conditions from each strain's dataset were used to define two patterns: equivalent (1111,1111) and differential (1111,2222) expression. The posterior probability for each pattern was calculated using a hierarchical log-normal normal expression model with the conditional false discovery rate (cFDR) at 0.01 to determine the appropriate threshold (cFD(τ)). The critical thresholds for the datasets were: 0.937 and 0.927 for comparison of strain GMI1000 expression between the two temperatures in culture and *in planta*, respectively; and 0.944 and 0.941 for comparison of UW551 expression between the two temperatures in culture and *in planta*, respectively. Data were imported into a custom MicroSoft Access database and further analyzed using Microsoft Excel; scatter plots were generated using ArrayStar to visualize expression data within and across treatments. The raw microarray data and MIAME information are available as Accession E-GEOD–33662 on the EMBL European Bioinformatics Institute ArrayExpress Archive (http://www.ebi.ac.uk/arrayexpress/).

### Quantitative RT-PCR (qPCR)

qPCR was performed as described [52] using an ABI PRISM 7300 Real-Time PCR System (Applied Biosystems, Foster, CA) with SYBR-Green chemistry. Based on observed stable expression in the microarrays under all conditions, three *R*. *solanacearum* genes, *oxyR*, *rplM*, and *serC*, were selected to normalize expression values for all other genes. The qPCR primer sequences are listed in S9 Table. Reaction parameters for qPCR were: 10 min polymerase activation, followed by 40 cycles of 95°C for 15 sec and 57°C for 1 min.

### Comparative genomics

Orthologous (homologous) proteins between genomes were identified using BLAST-based methods. Orthologs were assigned to genes whose similarity scores and percent identity fell within cutoff values of >70% for pair-wise BLASTP searches. Whole genome alignments using Mauve v2.1 [87] allowed us to use gene context as an arbiter in cases where multiple paralogs were possible and to designate orthologs with improved specificity and precision. When true orthology was critical, downstream analyses were limited to high confidence datasets.

### DNA manipulations

Cloning, restriction digestion, sequencing, and PCR were performed using standard methods [88]. *R*. *solanacearum* and *E*. *coli* were transformed by electroporation as previously described [89]. DNA sequencing and oligonucleotide synthesis were performed at the University of Wisconsin-Madison Biotechnology Center. DNA sequence was analyzed using the DNASTAR software package (DNASTAR, Inc., Madison, WI, U.S.A.). Unless otherwise noted, molecular biology reagents and kits were purchased from Promega (Madison, WI).

### Mutant strain construction

Splicing by overlap extension PCR (SOE-PCR) was used to create in-frame deletion constructs using primers listed in S9 Table [90]. Deletion constructs were introduced into the chromosome of wild-type *R*. *solanacearum* strain UW551 by double homologous recombination as previously described [38] to create UW551ΔlecM, UW551ΔaidA, UW551ΔaidC and UW551ΔsolI, respectively. The correct allelic replacement in each mutant was confirmed by PCR and sequencing analyses. To generate GMI1000 strains carrying the putative cool virulence region from UW551, the 2,534-bp DNA fragment containing the *lecM-aidA-aidC* region plus 753bp of upstream of *lecM* or a 4,149-bp fragment containing the *lecM-aidA-aidC-solR-solI* region were amplified from UW551 genomic DNA using the PCR primer pairs Up lecM_ F/Com aidC _R and ComWhole _F/ComWhole _R, respectively, and cloned into the *Sma*I site of pUC18-mini-Tn7T-Gm [91]. The resulting products were introduced into strain GMI1000 at the selectively neutral *att*Tn7 site 25bp downstream of the *glmS* gene [92] as described [93] and the correct integration was confirmed with PCR.

### Natural transformation of *R*. *solanacearum* strains

Natural transformation was used to make mutants in strains UW551Rif, UW553 and UW560 as previously described [94] with some modifications. Briefly, recipient strains were grown to mid-log phase in CPG, pelleted, washed and resuspended in sterile water. Fifty microliters of competent bacterial cells were incubated for 24h with 500 ng of genomic DNA from donor strains carrying the desired mutations, and transformants were selected on CPG plates containing the appropriate antibiotics. The mutants were confirmed by PCR and sequencing analyses.

### Virulence assays

To compare the virulence of *R*. *solanacearum* strains at different temperatures, we used a naturalistic soil soak inoculation and a more direct petiole inoculation of the wilt-susceptible tomato cv. Bonny Best, as previously described [95], except that half the plants were moved from 28°C to 20°C growth chambers one day after transplanting. For the soil soak assay, a bacterial suspension was poured onto the soil of pots that each contained an unwounded 16-day-old tomato plant to a final density of approximately 1x10^8^ cfu g^−1^ soil. For the petiole inoculation, 2x10^3^ bacteria in a 2μL volume were placed onto the freshly cut petiole of the first true leaf of a 21-day-old tomato plant. Plants were monitored daily for disease progress by a rater blind to treatment identity, and symptoms were scored on a 0-to–4 disease index, where 0 indicates no disease, 1 indicates 1 to 25% of leaves wilted, 2 indicates 26 to 50% of leaves wilted, 3 indicates 51 to 75% of leaves wilted, and 4 indicates 76 to 100% of leaves wilted. Each experiment contained a minimum of 16 plants per strain at each temperature, and experiments were repeated at least three times. The disease index for each day is the average of 48 plants from three experiments.

### Multiplication of *R*. *solanacearum* strains in tomato leaves

To ensure that mutant strains had not lost the ability to multiply in the host, we measured growth of strains uniformly infused into tomato leaves. *R*. *solanacearum* cells grown overnight in CPG were collected by centrifugation, resuspended in water, adjusted to OD_600_ = 0.01, and infiltrated into fully expanded tomato leaves with a syringe. Plants were incubated at either 28°C or 20°C. Every 12 hours for three days, leaves were sampled with a #5 cork borer, three leaf discs were pooled, ground, and dilution plated on TZC medium to enumerate colony forming units cm^−2^ leaf tissue. The experiment was repeated three times, with at least six plants per strain per temperature.

### Colonization of tomato stems by *R*. *solanacearum* strains

To measure bacterial colonization of tomato stems at 20°C, plants were inoculated with either the wild-type strain or the mutant using the cut petiole method as described above. Population sizes of each strain in tomato plants were quantified as described [38]. Briefly, at different time points after inoculation, five plants inoculated with each strain were randomly chosen and a 1 cm stem segment spanning the inoculation site was collected, weighed, and ground in 1 ml sterile deionized water. The resulting homogenate was dilution plated on TZC plates supplemented with 100 mg l^−1^ cycloheximide and appropriate antibiotics. *R*. *solanacearum* population sizes were determined as cfu g^−1^ of plant tissue. All experiments were repeated three times.

### Measuring bacterial attachment to tomato roots

Attachment of *R*. *solanacearum* cells to tomato seedling roots was quantified as described [80]. Briefly, susceptible tomato cv. ‘Bonny Best’ seedlings were grown axenically on Murashige and Skoog medium with vitamins (Caisson Laboratories, North Logan, UT) for 2 weeks, when secondary roots were well developed. These were incubated with *R*. *solanacearum* cell suspensions in water (OD_600_ = 0.01) at 20°C for 1h. For competition studies, wild type and mutant cell suspensions were combined in a 1:1 ratio. Seedling roots were then rinsed with sterile water, blotted lightly on absorbent paper, excised, weighed, ground in sterile water and dilution plated on TZC to quantify total bacteria adhering to roots. Cell numbers were normalized to seedling root fresh weight. All experiments were repeated three times, with each replicate containing at least 10 roots per treatment. For the sugar inhibition attachment assay, *R*. *solanacearum* cells were prepared as described above, except they were resuspended either in sterile water or water containing 0.01M D-mannose and 0.01M L-fucose, or 0.01M glucose as a control, and incubated at room temperature for half an hour. The bacterial suspensions were then incubated with sterile tomato seedlings at 20°C for an hour as described above.

### Biofilm assays

Production of biofilms by *R*. *solanacearum* strains was measured *in vitro* using a minor modification of the polyvinylchloride (PVC) microtiter plate assay [96]. Briefly, 5 μL overnight cultures of *R*. *solanacearum* adjusted to OD_600_ = 0.1 were used to inoculate 95 μL of CPG broth in wells of a PVC microtiter plate and incubated without shaking for 24h at 28°C. Biofilms were quantified by absorbance at 600nm following crystal violet staining.

### Survival in potato tubers

We measured the survival of *R*. *solanacearum* strains in susceptible 15 to 17 mm potato minitubers (cv. ‘Russet Norkotah’, Sklarczyk Seed Farm, Johannesburg, MI) at the typical seed potato storage temperature of 4°C as previously described [9]. Briefly, both ends of each potato tuber were injected with 2 μL of 1x10^9^ cfu ml^−1^ bacterial suspensions. Tubers were stored in the dark at 4°C and sampled at intervals. Tissue was weighed, ground, and dilution plated to determine cfu g^−1^. Survival rates were calculated based on triplicate experiments using two to three individual tubers per sampling time point. The detection limit was 1 log cfu g^−1^ potato tissue and a value of 1 log cfu g^−1^ was also used to report samples below the detection limit. Experiments were performed with rifampicin-resistant *R*. *solanacearum* strains to facilitate pathogen detection in the natural microbial background.

## Supporting Information

S1 Fig Scatter plots of expression levels of all *R*. *solanacearum* UW551 and GMI1000 at 20°C and 28°C during tomato pathogenesis. Within each strain, gene expression was highly correlated at temperate and tropical temperatures. Scatter plots showing *in planta* mean signal intensities of genes in the genomes of UW551 (A) and GMI1000 (B) at 20°C and 28°C, as determined by whole-genome microarray analysis. Each dot represents a gene, and the log2 signal intensity for each gene shown is the average of four biological replicates.
(PDF)

S2 Fig Venn diagrams of *R*. *solanacearum* UW551 and GMI1000 genes differentially expressed by temperature in culture and during tomato pathogenesis. A small number of genes were differentially expressed (DE) at 20°C compared to 28°C, with minimal overlap between those DE in culture and *in planta*. The numbers in the circles indicate the number of genes DE under different conditions (>2-fold difference in expression by EBArray analysis with the false discovery rate set at 0.01).
(PDF)

S3 Fig The *R*. *solanacearum* hydroxycinnamic acid degradation pathway and effect of cool temperature on its expression. The hydroxycinnamic acid degradation pathway was up-regulated in *R*. *solanacearum* strain GMI1000 during tomato pathogenesis at 20°C. Above each arrow is shown the gene(s) encoding each enzyme, together with their expression level fold-changes at 20°C compared to 28°C *in planta* are shown above the arrows.
(PDF)

S4 Fig Virulence effects of mutating *lecM*, *aidA*, and *aidC* in two additional R3bv2 *R*. *solanacearum* strains. Mutation of *lecM*, *aidA*, or *aidA* in *R*. *solanacearum* R3bv2 strains UW553 (A) and UW560 (B) resulted in significantly lower bacterial wilt virulence than the corresponding wild-type parent strain at 20°C (*P*< 0.01 by repeated measures ANOVA). Virulence was measured on wilt-susceptible tomato plants at 20°C via soil soak inoculation. The experiment was repeated twice, each replicate containing 16 plants per treatment per strain. Results from a representative experiment are shown.
(PDF)

S1 Table qPCR validation of expression levels for some *R*. *solanacearum* strain UW551 genes differentially expressed in the microarray analysis. (PDF)

S2 Table *R*. *solanacearum* strain UW551 genes differentially expressed *in planta* at 20°C compared to 28°C. (PDF)

S3 Table *R*. *solanacearum* strain UW551 genes differentially expressed in rich culture medium (CPG) at 20°C compared to 28°C. (PDF)

S4 Table *R*. *solanacearum* strain GMI1000 genes differentially expressed *in planta* at 20°C compared to 28°C. (PDF)

S5 Table *R*. *solanacearum* strain GMI1000 genes differentially expressed in rich culture medium (CPG) at 20°C compared to 28°C. (PDF)

S6 Table Genes involved in the salicylic acid degradation pathway were selectively up-regulated in *R*. *solanacearum* strain GMI1000 during tomato pathogenesis at 20°C. (PDF)

S7 Table Genes involved in multidrug efflux were up-regulated in *R*. *solanacearum* strain GMI1000 during tomato pathogenesis at 20°C. (PDF)

S8 Table Bacterial strains used in this study. (PDF)

S9 Table Sequences of PCR primers used in this study. (PDF)
