# Supplementary material for: Comparative Transcriptome Analysis Reveals Cool Virulence Factors of Ralstonia solanacearum Race 3 Biovar 2
Source: PLoS One. 2015 Oct 7;10(10):e0139090. doi: 10.1371/journal.pone.0139090 (PMC4596706; doi:10.1371/journal.pone.0139090)
Supplement: S1 Fig — Within each strain, gene expression was highly correlated at temperate and tropical temperatures. Scatter plots showing in planta mean signal intensities of genes in the genomes of UW551 (A) and GMI1000 (B) at 20°C and 28°C, as determined by whole-genome microarray analysis. Each dot represents a gene, and the log2 signal intensity for each gene shown is the average of four biological replicates. (PDF) [file pone.0139090.s001.pdf]

S1 Figure. Scatter plots of expression levels of all *R. solanacearum* UW551 and GMI1000 ORFs at 20°C and 28°C during tomato pathogenesis.

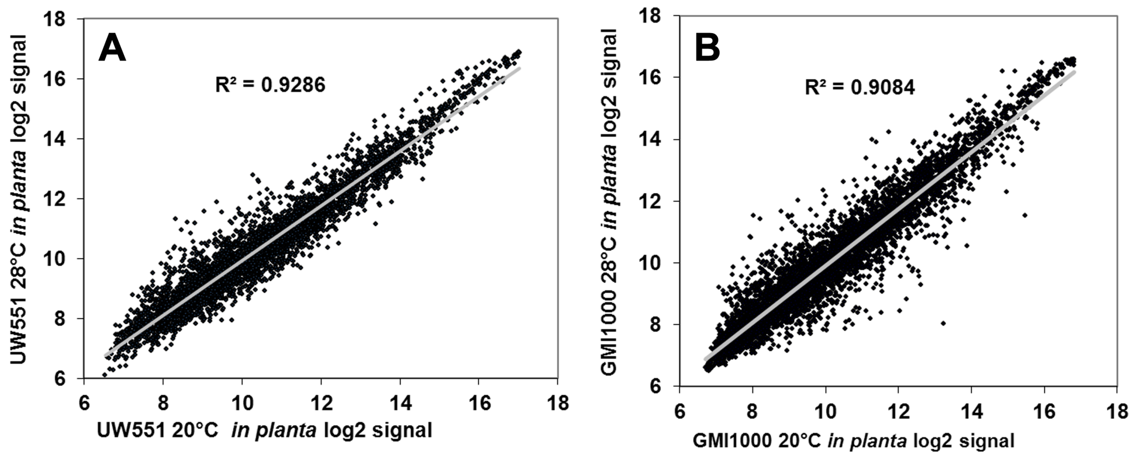

S1 Figure. Scatter plots of expression levels of all *R. solanacearum* UW551 and GMI1000 ORFs at 20°C and 28°C during tomato pathogenesis. Within each strain, gene expression was highly correlated at temperate and tropical temperatures. Scatter plots show *in planta* mean signal intensities of genes in the genomes of UW551 (A) and GMI1000 (B) at 20°C and 28°C, as determined by whole-genome microarray analysis. Each dot represents a gene, and the log2 signal intensity for each gene shown is the average of four biological replicates.
