# Supplementary material for: Comparative Transcriptome Analysis Reveals Cool Virulence Factors of Ralstonia solanacearum Race 3 Biovar 2
Source: PLoS One. 2015 Oct 7;10(10):e0139090. doi: 10.1371/journal.pone.0139090 (PMC4596706; doi:10.1371/journal.pone.0139090)
Supplement: S2 Fig — A small number of genes were differentially expressed (DE) at 20°C compared to 28°C, with minimal overlap between those DE in culture and in planta. The numbers in the circles indicate the number of genes DE under different conditions (>2-fold difference in expression by EBArray analysis with the false discovery rate set at 0.01). (PDF) [file pone.0139090.s002.pdf]

S2 Figure. Venn diagrams of *R. solanacearum* UW551 and GMI1000 genes differentially expressed by temperature in culture and during tomato pathogenesis.

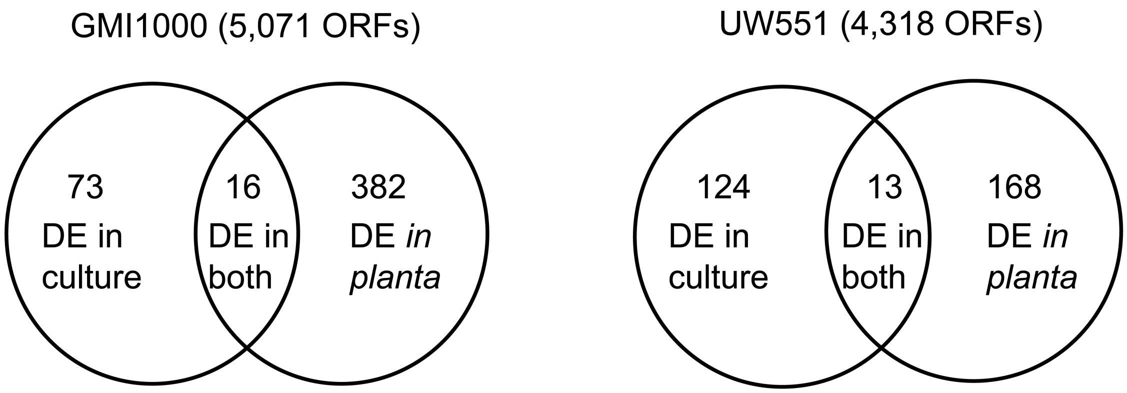

S2 Figure. Venn diagrams of *R. solanacearum* UW551 and GMI1000 genes differentially expressed by temperature in culture and during tomato pathogenesis. A small number of genes were differentially expressed (DE) at 20°C compared to 28°C, with minimal overlap between the genes that were DE in culture and *in planta*. The numbers in the circles indicate the number of genes DE under different conditions (>2-fold difference in expression by EBArray analysis with the false discovery rate set at 0.01).
