# Supplementary material for: Comparative Transcriptome Analysis Reveals Cool Virulence Factors of Ralstonia solanacearum Race 3 Biovar 2
Source: PLoS One. 2015 Oct 7;10(10):e0139090. doi: 10.1371/journal.pone.0139090 (PMC4596706; doi:10.1371/journal.pone.0139090)
Supplement: S3 Fig — The hydroxycinnamic acid degradation pathway was up-regulated in R. solanacearum strain GMI1000 during tomato pathogenesis at 20°C. Above each arrow is shown the gene(s) encoding each enzyme, together with their expression level fold-changes at 20°C compared to 28°C in planta are shown above the arrows. (PDF) [file pone.0139090.s003.pdf]

S3 Figure. The *R. solanacearum* hydroxycinnamic acid degradation pathway and effect of cool temperature on its expression.

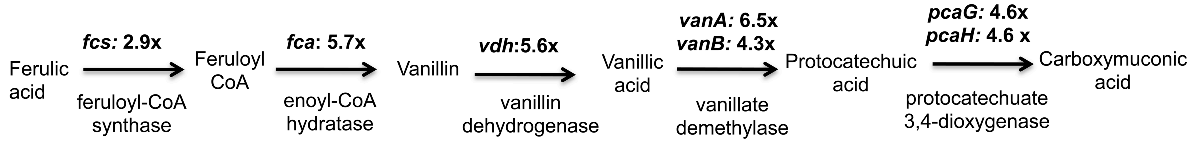

S3 Figure. The *R. solanacearum* hydroxycinnamic acid degradation pathway and effect of cool temperature on its expression. The hydroxycinnamic acid degradation pathway was up-regulated in *R. solanacearum* strain GMI1000 during tomato pathogenesis at 20°C. Above each arrow is shown the gene(s) encoding each enzyme, together with their expression level fold-changes at 20°C compared to 28°C *in planta*.
