# Supplementary material for: Comparative Transcriptome Analysis Reveals Cool Virulence Factors of Ralstonia solanacearum Race 3 Biovar 2
Source: PLoS One. 2015 Oct 7;10(10):e0139090. doi: 10.1371/journal.pone.0139090 (PMC4596706; doi:10.1371/journal.pone.0139090)
Supplement: S4 Fig — Mutation of lecM, aidA, or aidA in R. solanacearum R3bv2 strains UW553 (A) and UW560 (B) resulted in significantly lower bacterial wilt virulence than the corresponding wild-type parent strain at 20°C (P< 0.01 by repeated measures ANOVA). Virulence was measured on wilt-susceptible tomato plants at 20°C via soil soak inoculation. The experiment was repeated twice, each replicate containing 16 plants per treatment per strain. Results from a representative experiment are shown. (PDF) [file pone.0139090.s004.pdf]

**S4 Figure. Virulence effects of mutating *lecM, aidA*, and *aidC* in two additional R3bv2 *R. solanacearum* strains.**

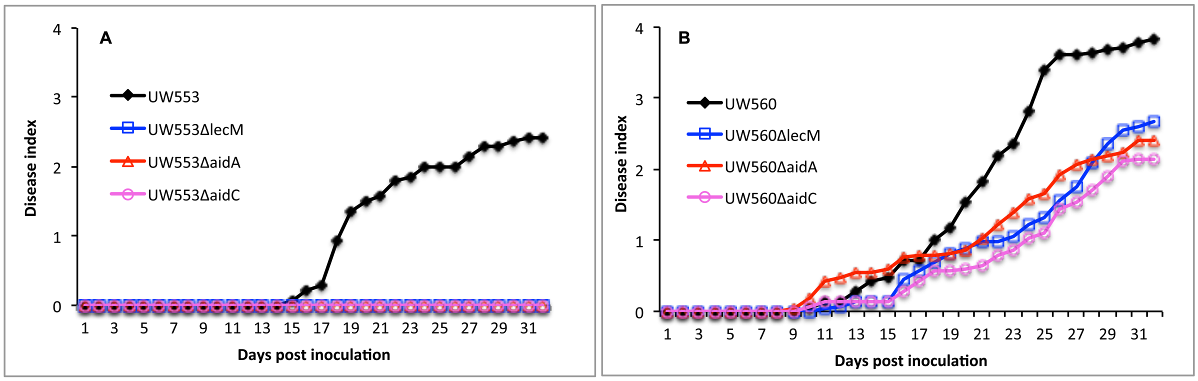


**S4 Figure. Virulence effects of mutating *lecM, aidA*, and *aidC* in two additional R3bv2 *R. solanacearum* strains.** Mutation of *lecM*, *aidA*, or *aidA* in *R. solanacearum* R3bv2 strains UW553 (A) and UW560 (B) resulted in significantly lower bacterial wilt virulence than the corresponding wild-type parent strain at 20°C ($P$<0.01 by repeated measures ANOVA). Virulence was measured on wilt-susceptible tomato plants at 20°C via soil-soak inoculation. The experiment was repeated twice, each replicate containing 16 plants per treatment per strain. Results from a representative experiment are shown.
