# Supplementary material for: Comparative Transcriptome Analysis Reveals Cool Virulence Factors of Ralstonia solanacearum Race 3 Biovar 2
Source: PLoS One. 2015 Oct 7;10(10):e0139090. doi: 10.1371/journal.pone.0139090 (PMC4596706; doi:10.1371/journal.pone.0139090)
Supplement: S1 Table — (PDF) [file pone.0139090.s005.pdf]

S1 Table. qPCR validation[a] of expression levels for some *R. solanacearum* strain UW551 genes differentially expressed in the microarray analysis.

Gene name	Locus tag	Array 20°C/28°C fold-change[b]	qPCR1 20°C/28°C fold-change[b]	qPCR2 20°C/28°C fold-change[b]	qPCR3 20°C/28°C fold-change[b]	Average qPCR 20°C/28°C fold-change[2]
lecM	RRSL_02788	7.19	39.83	40.88	32.45	37.72
aidA	RRSL_02789	9.58	31.6	35.42	34.56	33.86
aidC	RRSL_02790	3.55	23.32	30.46	27.24	27.01
solR	RRSL_02791	2.4	2.81	2.46	2.47	2.58
n/a[c]	RRSL_03846	-3.1	-1.76	-1.54	-1.19	-1.5
n/a[c]	RRSL_0475	-4.35	-2.83	-2.72	-2.36	-2.64

[a]RNA was extracted from UW551 cells grown in rich CPG broth to log phase ($6x10^8$ CFU/mL) either at 28°C or 20°C, and reverse transcribed into cDNA for qPCR analysis.

[b]Fold change numbers reflect the relative expression of each gene at 20°C relative to its expression at 28°C. Expression levels of *oxyR*, *rplM*, and *serC* were used to normalize the expression values of other genes under each condition.

[c]*n/a*, no gene name assigned. RRSL_03846 is predicted to encode a cold shock protein and RRSL_0475 is predicted to encode an enzyme involved in trehalose synthesis.
