# Supplementary material for: Comparative Transcriptome Analysis Reveals Cool Virulence Factors of Ralstonia solanacearum Race 3 Biovar 2
Source: PLoS One. 2015 Oct 7;10(10):e0139090. doi: 10.1371/journal.pone.0139090 (PMC4596706; doi:10.1371/journal.pone.0139090)
Supplement: S2 Table — (PDF) [file pone.0139090.s006.pdf]

S2 Table. *R. solanacearum* strain UW551 genes differentially expressed *in planta* at 20°C compared to 28°C.

Gene symbol	Fold-change[a]	UW551 locus tag	GMI1000 locus tag[b]	Gene product
hrcC	2.47	RRSL_00024	RSp0874	HrcC
	2.36	RRSL_00026		Enoyl-[acyl-carrier-protein] reductase (NADH) (EC:1.3.1.9)
	2.47	RRSL_00027		Probable acetate kinase
	2.95	RRSL_00029		Hypothetical protein
phnS2	-2.57	RRSL_00130		Putative 2-aminoethylphosphonate ABC transporter, periplasmic 2-aminoethylphosphonate-binding protein
	-2.39	RRSL_00193	RS04714	Hypothetical Protein
	-2.42	RRSL_00201		Phage protein
	-2.6	RRSL_00220		Hypothetical protein
	2.65	RRSL_00246	RS03668	Hypothetical Protein
dppC	-2.41	RRSL_00275		DppC
	-2.81	RRSL_00281		Aldehyde dehydrogenase (EC:1.2.1.3)
	-3.22	RRSL_00282		Aldehyde dehydrogenase (EC:1.2.1.3)
	2.21	RRSL_00314		Tryptophan synthase beta chain (EC:4.2.1.20)
	-2.29	RRSL_00355		C-5 sterol desaturase (EC:1.3.-)
	-2.32	RRSL_00360		Hypothetical Protein
	-2.5	RRSL_00375	RS03190	Translation initiation inhibitor
	-2.56	RRSL_00397		Hypothetical protein
	-2.67	RRSL_00406	RSc0655	GspD-related protein; cpaC1
	-2.9	RRSL_00409	RSc0657	Prepilin Peptidase; cpaA1
	-2.48	RRSL_00410	RSc0660	Putative Pilin Protein
	-2.39	RRSL_00423	RSc1856	Hypothetical Protein
	2.28	RRSL_00426	RSc1386	Conserved Hypothetical Protein
	2.57	RRSL_00443		Homoserine O-acetyltransferase (EC:2.3.1.31)
	-2.67	RRSL_00478	RS02975	Hypothetical Protein
hrpK	2.71	RRSL_00518	RSp0865	HrpK
hrpH	2.43	RRSL_00520	RSp0868	HrpH
	2.97	RRSL_00529	RSc1802	Transcriptional regulator, AraC family
	3.08	RRSL_00530		Hypothetical protein
	-3.55	RRSL_0054[c]	RSc1793	ABC transporter permease protein
	-2.52	RRSL_00542[c]	RSc1792	Hypothetical Protein
	-4.57	RRSL_00585	RSp1397	3-dehydroquinate dehydratase (EC:4.2.1.10)
	-2.83	RRSL_00587	RS02059	Outer membrane porin protein 32 precursor
	-2.36	RRSL_00599	RSc2284	Outer membrane protein
livH	-3.48	RRSL_00626	RS01986	LivH
livM	-3.1	RRSL_00627	RS01987	LivM
	2.75	RRSL_00645	RSc3215	Hypothetical Protein
	2.55	RRSL_00704	RSc1474	Hypothetical Protein
	-3.74	RRSL_00712		Muconate cycloisomerase (EC:5.5.1.1)
	-2.34	RRSL_00727	RSc2726	Transmembrane multidrug resistance transport protein. HLYD family
	-2.92	RRSL_00727_1[c]	RSc2727	Multidrug resistance protein, MFS family
	-2.84	RRSL_00728[c]	RSc2728	Hypothetical protein
	-2.28	RRSL_00804	RSc3341	LivH
	-2.69	RRSL_00806	RSc3340	LivM
	-2.24	RRSL_00837		Transcriptional regulator

	-6.01	RRSL_00840		Hypothetical Protein
	-2.46	RRSL_00844[c]	RSc3382	Potassium-transporting ATPase A chain (EC:3.6.3.12)
	-2.4	RRSL_00858	RSc3363	2',3'-cyclic-nucleotide 2'-phosphodiesterase / 3'-nucleotidase (EC:3.1.3.6,EC:3.1.4.16)
	-2.23	RRSL_00908	RSc1080	Hexuronate transporter
	-4.88	RRSL_00910[c]	RSc1079	Glucarate dehydratase (EC:4.2.1.40)
	-4.43	RRSL_00945	RS05465	Hypothetical Protein
	2.29	RRSL_00949	RS05473	Acetyltransferase (EC:2.3.1.-)
	2.95	RRSL_00950	RS05475	Hypothetical Protein
	2.96	RRSL_01022	RS05358	Hypothetical Protein
	2.36	RRSL_01034	RS05370	Spermidine synthase (EC:2.5.1.16)
	-2.33	RRSL_01121	RSp1224	Probable aerotaxis receptor (aer1)
	-3.46	RRSL_01144		Hypothetical Protein
	3.34	RRSL_01223	RSc2561	Hypothetical Protein
	3.27	RRSL_01250		Hypothetical Protein
	2.95	RRSL_01251		Hypothetical Protein
	2.74	RRSL_01265	RSc3201	Dehydrogenase protein DltE
	2.31	RRSL_01284[c]	RSc2151	Hypothetical Protein
	-2.31	RRSL_01301	RSc1737	Outer membrane porin protein 32 precursor
	-2.54	RRSL_01306[c]	RSc1732	ABC transporter permease protein
	-2.59	RRSL_01348	RSc1381	DppB
	-2.31	RRSL_01367		Bile acid-inducible operon protein F
	-2.37	RRSL_01462		Hypothetical Protein
	-6.35	RRSL_01507	RSc0760	Metal-dependent hydrolase
	-2.89	RRSL_01525	RSc0780	Long-chain-fatty-acid--CoA ligase (EC:6.2.1.3)
	-2.37	RRSL_01540	RSc1336	Sulfate-binding protein
	-2.28	RRSL_01541	RSc1337	FMN reductase (EC:1.5.1.29)
	-6.11	RRSL_01542		Alkanesulfonates-binding protein
	-3.08	RRSL_01545	RSc1340	Alkanesulfonates transport system permease protein
	-2.22	RRSL_01556	RSc1351	Two-component sensor histidine kinase (EC:2.7.3.-)
	-4.63	RRSL_01610[c]	RSc1772	Acetyl-hydrolase (EC:3.1.1.-)
	-2.74	RRSL_01618[c]	RSc1783	Hypothetical protein
	-2.46	RRSL_01622		Hypothetical Protein
	-4.73	RRSL_01632	RSc0312	Biotin operon repressor / Biotin--[acetyl-CoA-carboxylase] synthetase (EC:6.3.4.15)
	-2.51	RRSL_01646		Fumarylacetoacetase (EC:3.7.1.2)
	-2.29	RRSL_01698	RS03722	Hemin receptor
	2.32	RRSL_01704[c]	RS03717	Hypothetical Protein
	2.47	RRSL_01749		Atypical orf
fenI	2.45	RRSL_01750	RS01950	FenI
	-2.28	RRSL_01820	RSp0508	Bacteriophage N4 adsorption protein B
	-3.15	RRSL_01910	RSc1166	Tetratricopeptide repeat family protein
	-5.48	RRSL_01938	RSc1139	Ferredoxin--NAD(P)(+) reductase (EC:1.18.1.-)
	-2.72	RRSL_01975	RSc2860	Transcriptional regulatory protein
	-3	RRSL_02004	RSc0075	16S rRNA m(5)C 967 methyltransferase (EC:2.1.1.-)
	2.29	RRSL_02060		Drug/metabolite transporter (DMT) permease
	-2.21	RRSL_02145	RSc1233	Cytosine/uracil/thiamine/allantoin permease family protein
	-2.37	RRSL_02159		Hypothetical protein
	-2.58	RRSL_02239		Hypothetical Protein
	-2.45	RRSL_02259		Hypothetical Protein
	-2.6	RRSL_02268	RSp0676	5-methyltetrahydropteroyltriglutamate-- homocysteine methyltransferase (EC:2.1.1.14)
	-2.37	RRSL_02306		Hypothetical Protein

	2.35	RRSL_02353		Hypothetical Protein
	2.6	RRSL_02354	RSc2703	Hypothetical Protein
	3.65	RRSL_02356		Hypothetical Protein
	-6.01	RRSL_02385	RSc0804	Transcriptional regulators, LysR family
	2.24	RRSL_02393	RSc0814	Transmembrane threonine efflux protein
	2.21	RRSL_02396	RSc0816	Hypothetical Protein
popC	2.49	RRSL_02441	RSp0875	PopC
	2.43	RRSL_02445		Conserved hypothetical protein
yaaA	-3.72	RRSL_02473	RSc2009	YaaA
	-3.01	RRSL_02517		Hypothetical protein
	-2.53	RRSL_02556	RSc0223	Hypothetical protein
	2.6	RRSL_02577		Conserved hypothetical protein
	2.23	RRSL_02578		Putative chromosome segregation ATPases
	2.25	RRSL_02579		Hypothetical protein
	2.36	RRSL_02611		Putative resolvase
	-4.42	RRSL_02656	RSc2802	Transporter, drug/metabolite exporter family
	-2.24	RRSL_02659[c]	RSc2798	Hypothetical Protein
	-2.7	RRSL_02730[c]	RSc2894	Hypothetical Protein
	2.22	RRSL_02760	RSc2926	Stringent starvation protein A
lecM	3.66	RRSL_02788	RSc3288	Mannose/fucose-binding lectin
aidA	3.52	RRSL_02789		AidA
	-3.04	RRSL_02805	RSc3276	Transcriptional regulator, GntR family
	-3.85	RRSL_02806[c]	RSc3275	C4-dicarboxylate transport protein
	2.28	RRSL_02815[c]	RSc3267	Rare lipoprotein A
	2.27	RRSL_02836		Usf protein
livM	-3.61	RRSL_02841		LivM
	2.48	RRSL_02853	RSp1279	Fumarate hydratase class II (EC:4.2.1.2)
	-2.49	RRSL_02877	RSc0216	Bicyclomycin resistance protein
	-2.22	RRSL_02898		Cyclohexanone monooxygenase (EC:1.14.13.22)
	-2.99	RRSL_02899[c]	RSc3079	Hypothetical protein
	-4.37	RRSL_02911		Hypothetical protein
	-2.79	RRSL_02925		Hypothetical protein
	-2.68	RRSL_03004		Amino acid ABC transporter permease protein
	-5.99	RRSL_03008		Hypothetical Protein
	2.76	RRSL_03036	RS00884	Hypothetical Protein
	-2.55	RRSL_03049		Oxidoreductase (EC:1.1.1.-)
	-2.73	RRSL_03050		Transcriptional regulators, LysR family
	3.34	RRSL_03101		Lactoylglutathione lyase
hutC	-8.43	RRSL_03201	RSc2648	Histidine utilization repressor
	-3.06	RRSL_03218	RSc2663	Cobalamin adenosyltransferase family protein
	-2.51	RRSL_03420		Hypothetical Protein
	-2.83	RRSL_03506	RSc2218	NAD(FAD)-utilizing dehydrogenases
	-3.08	RRSL_03534	RSc2243	Transcriptional regulators, LysR family
glnP	-2.44	RRSL_03551	RSc2258	GlnP
	-3.57	RRSL_03649		Transcriptional regulators, LysR family
	4.42	RRSL_03672	RSc3309	Glutathione S-transferase (EC:2.5.1.18)
	-3.83	RRSL_03693	RSc3331	Short chain dehydrogenase
	-2.5	RRSL_03696	RSc3335	NADPH-dependent glutamate synthase beta chain and related oxidoreductases
	-2.6	RRSL_03707	RSp0222	Vanillate O-demethylase oxygenase subunit (EC:1.14.13.82,EC:1.2.3.12)
	-2.59	RRSL_03710	RSp0225	Hypothetical Protein
	-2.71	RRSL_03714	RS05195	Transkriptions-aktivator-protein
	-3.11	RRSL_03718	RS05192	Hypothetical Protein

	-2.89	RRSL_03719	RS05191	Lipase (EC:3.1.1.3)
	2.3	RRSL_03808	RSc0476	dbi-related protein 1
	-2.31	RRSL_03869[c]	RSc2083	UDP-2,3-diacylglucosamine hydrolase (EC:3.6.1.-)
	2.42	RRSL_03919		Transcriptional regulators, LysR family
	2.24	RRSL_03920		Hypothetical Protein
	2.33	RRSL_03921	RSc1839	Shikimate kinase (EC:2.7.1.71)
	2.47	RRSL_03933		(partial?) integrase
	-2.46	RRSL_03968[c]	RSc3137	Hypothetical protein
	-2.55	RRSL_04130	RSc0546	Hypothetical Protein
	-2.6	RRSL_04146		Long-chain-fatty-acid--CoA ligase (EC:6.2.1.3)
	-2.36	RRSL_04174[c]	RSc3395	Hypothetical Protein
nhaC	-3.66	RRSL_04244		NhaC
	-2.22	RRSL_04314	RSc0155	Hypothetical cytosolic protein
	-3.03	RRSL_04322[c]	RSc0147	Hypothetical Protein
	-2.97	RRSL_04326	RSc0143	Hypothetical Protein
	-2.51	RRSL_04390	RSc1015	RbsC
	-2.85	RRSL_04410	RSc0993	Transcriptional regulator, ArsR family
	2.53	RRSL_04505		Hypothetical protein
	2.4	RRSL_04506		Hypothetical protein
	2.69	RRSL_04508		Hypothetical protein
	2.68	RRSL_04512[c]	RSc1586	Hypothetical protein
	-2.92	RRSL_04535	RSc1610	Transcriptional regulator, IclR family
	-2.25	RRSL_04581		Hypothetical protein
	2.26	RRSL_04598	RSp0978	Molybdopterin-guanine dinucleotide biosynthesis protein B
	-2.25	RRSL_04617	RS03033	ABC transporter permease protein
	-2.8	RRSL_04624	RS04790	Hypothetical Protein
	2.35	RRSL_04659		Hypothetical Protein
	-3.01	RRSL_04694	RS05071	Transmembrane type 1 secretion system outer membrane efflux protein
	-2.64	RRSL_04696		Hypothetical Protein
	-2.43	RRSL_04724		Hypothetical Protein
modB	-3.35	RRSL_04731[c]	RS05467	ModB
	2.6	RRSL_04734[c]	RS05474	Hypothetical Protein
hrpB	2.33	RRSL_10003[c]	RSp0873	HrpB
	-2.59	RRSL_RNA027		(tRNA)
	-2.85	RRSL_RNA038		(tRNA)

[a]Fold change was calculated based on gene expression at 20°C compared to 28°C *in planta.* Positive values indicate up-regulation of genes at 20°C, and negative values indicate down-regulation of genes at 20°C.
[b] The GMI1000 locus tag was shown if the corresponding ortholog in GMI1000 exists.
[c] Indicates genes from *R. solanacearum* species complex core genome that were differentially expressed by temperature *in planta* in both strains.
