# Supplementary material for: Comparative Transcriptome Analysis Reveals Cool Virulence Factors of Ralstonia solanacearum Race 3 Biovar 2
Source: PLoS One. 2015 Oct 7;10(10):e0139090. doi: 10.1371/journal.pone.0139090 (PMC4596706; doi:10.1371/journal.pone.0139090)
Supplement: S3 Table — (PDF) [file pone.0139090.s007.pdf]

S3 Table. *R. solanacearum* strain UW551 genes differentially expressed in rich culture medium (CPG) at 20°C compared to 28°C.

Gene symbol	Fold-change[a]	UW551 locus tag	GMI1000 Locus tag[b]	Gene product
	-2.18	RRSL_00067	RS03728	Peptide synthetase
	-3.04	RRSL_00121	RSp0239	1,4-alpha-glucan branching enzyme (EC:2.4.1.18)
	-2.52	RRSL_00122	RS05183	Trehalose synthase
	2.01	RRSL_00168	RS01956	Hypothetical protein
	2.44	RRSL_00256		Hypothetical protein
	2.01	RRSL_00419		Hypothetical protein
hrcT	2.64	RRSL_00525	RSp0872	HrcT
	2.12	RRSL_00530		Hypothetical protein
	-2.25	RRSL_00545	RSc1789	Hypothetical protein
	3.57	RRSL_00575	RS02288	Hypothetical protein
	-2.02	RRSL_00616	RSp0006	UTP--glucose-1-phosphate uridylyltransferase (EC:2.7.7.9)
	-2.76	RRSL_00744		Porin
	-2.36	RRSL_00745	RS02001	Hypothetical protein
	2.18	RRSL_00754	RSc0741	Probable chemotaxis response regulator protein (cheY2)
	-2.5	RRSL_00833		Hypothetical protein
	3	RRSL_00840		Hypothetical protein
	-2.8	RRSL_00920	RS05439	Molybdopterin-guanine dinucleotide biosynthesis protein A
	-4.1	RRSL_00921	RS05440	Hypothetical protein
mexC	2.34	RRSL_00937	RSp0313	MexC
mexD	2.43	RRSL_00938	RSp0312	MexD
	2.47	RRSL_00939	RSp0311	Type I secretion outer membrane protein
	-2.02	RRSL_00977	RSc2358	Phosphoenolpyruvate carboxylase (EC:4.1.1.31)
	2.93	RRSL_01069		Hypothetical protein
	2.48	RRSL_01070		Hypothetical protein
	2.69	RRSL_01114		Hypothetical protein
	2.59	RRSL_01249		Adenine DNA methyltransferase-like protein
	2.26	RRSL_01250		Hypothetical protein
	3.64	RRSL_01251		Hypothetical protein
	-2.04	RRSL_01301	RSc1737	Outer membrane porin protein 32 precursor
	3.91	RRSL_01308	RSc1730	Short-chain type dehydrogenase/reductase (EC:1.-)
	3.13	RRSL_01309	RSc1729	Transcriptional regulators, LysR family
	3.24	RRSL_01310	RSc1728	Hypothetical protein
	2.4	RRSL_01312	RSc1726	Hypothetical protein
	2.6	RRSL_01313	RSc1725	Hypothetical protein
	-2.06	RRSL_01342		Membrane-fusion protein (Cell envelope biogenesis, outer membrane)
	-2.02	RRSL_01364		Molybdopterin biosynthesis MoeB protein
	-2.72	RRSL_01365		MEC+
	-2.6	RRSL_01366		ThiS family protein
	-2.25	RRSL_01367		Bile acid-inducible operon protein F
	-2.13	RRSL_01368		2,3-dihydroxybenzoate-AMP ligase (EC:2.7.7.58)
	-2.16	RRSL_01369		Acyl-CoA dehydrogenase (EC:1.3.99.-)
	-2.14	RRSL_01372		Hypothetical protein

	-2.12	RRSL_01373		Possible TonB-dependent receptor
	-2.37	RRSL_01387		Hypothetical protein
	2.17	RRSL_01401	RSc1276	Cytochrome c oxidase polypeptide I (EC:1.9.3.1)
	-5.56	RRSL_01417		Hypothetical protein
	-2.05	RRSL_01451	RSc2526	Superoxide dismutase (EC:1.15.1.1)
	-4.91	RRSL_01475	RSc2502	Hypothetical protein
	-2.44	RRSL_01476		Hypothetical protein
	-2.37	RRSL_01539	RSc1335	ClpB protein
glgA	-3.82	RRSL_01693	RSp0242	Probable glycogen synthase, glgA (EC:2.4.1.21)
	-2.2	RRSL_01826	RS05802	Hypothetical protein
	-2.65	RRSL_01827	RS00392	Mechanosensitive ion channel
	2.29	RRSL_02047		Hypothetical protein
	2.02	RRSL_02048		Hypothetical protein
	3.99	RRSL_02063		Hypothetical protein
	2.77	RRSL_02066		Hypothetical protein
ptsA	2.06	RRSL_02178	RSc1531	PstA
	2.98	RRSL_02354	RSc2703	Hypothetical protein
	3.08	RRSL_02355		Bacterial protein Translation Initiation Factor 1 (IF-1)
	5.11	RRSL_02356		Hypothetical protein
	-2.17	RRSL_02512		Putative osmotically inducible lipoprotein B1 transmemberane-related protein
	-2.08	RRSL_02560		Conserved Hypothetical protein
	2.24	RRSL_02625		Hypothetical protein
	-2.36	RRSL_02654	RSc2803	Hypothetical protein
	2.38	RRSL_02785	RSc3292	Integral membrane protein
lecM	7.19	RRSL_02788	RSc3288	Mannose/ fucose-binding lectin
aidA	9.58	RRSL_02789		AidA
aidC	3.55	RRSL_02790		Hypothetical protein
solR	2.4	RRSL_02791	RSc3287	SolR
solI	2.09	RRSL_02792	RSc3286	SolI
	-3.49	RRSL_02891	RSc3083	Hypothetical protein
	-2.6	RRSL_02892		Hypothetical protein
	-3.79	RRSL_02923	RSc3053	Hypothetical protein
	-2.43	RRSL_02955	RSc0023	Hypothetical protein
	-2.16	RRSL_03027	RS00875	Ornithine cyclodeaminase (EC:4.3.1.12)
	-2.47	RRSL_03030	RS00878	Hypothetical IucA protein (EC:6.-)
	-2.51	RRSL_03031	RS00879	Hypothetical IucC protein (EC:6.-)
	-2.05	RRSL_03032	RS00880	4-hydroxy-2-oxovalerate aldolase (EC:4.1.2.)
	-3.15	RRSL_03038	RS02188	Cyanate lyase (EC:4.2.1.104,EC:4.3.99.1)
	2.75	RRSL_03068	RS02205	Hypothetical protein
	-2.21	RRSL_03084	RS02224	Hypothetical protein
	-2.85	RRSL_03085	RS02225	Hypothetical protein
	-2.53	RRSL_03086	RS02226	Two-component response regulator
	-4.35	RRSL_03089	RS02229	Hypothetical protein
rpoN	-2.11	RRSL_03090	RSp1671	RpoN
	-2.27	RRSL_03093	RS02233	Hypothetical protein
	-2.75	RRSL_03094	RS02235	Hypothetical protein
	-2.91	RRSL_03096		Hypothetical protein
nac	-2.38	RRSL_03134	RSp0942	Nac
	-2.38	RRSL_03135	RSp0941	Hypothetical protein
	2.26	RRSL_03161	RS02477	Probable hemagglutinin-related protein
	-3.81	RRSL_03167	RS02580	NAD-dependent oxidoreductase
	-2.69	RRSL_03184	RS02600	putative hemagglutinin-related transmembrane

				protein
	-2.15	RRSL_03321	RSc0341	Hypothetical protein
	2.07	RRSL_03353		Hypothetical protein
	2.18	RRSL_03366	RS02393	Omega-amino acid--pyruvate aminotransferase (EC:2.6.1.18)
	-2.15	RRSL_03395	RSp0122	Methylisocitrate lyase (EC:4.1.3.30)
	2.68	RRSL_03527	RSc2238	Hypothetical protein
	2.07	RRSL_03700		Hypothetical protein
glgX	-2.21	RRSL_03726	RSp0238	GlgX
	-3.48	RRSL_03776	RSc0443	Hypothetical protein
	-2.15	RRSL_03777	RSc0444	Hypothetical protein
	2.09	RRSL_03828		Hypothetical protein
	-3.1	RRSL_03846	RSc2466	Cold shock protein
	2.17	RRSL_03922		Hypothetical protein
	2.27	RRSL_03923		Hypothetical protein
	2.11	RRSL_03933		(partial?) integrase
	-3.32	RRSL_03978	RSc3128	Aldehyde dehydrogenase (EC:1.2.1.3)
	-2.22	RRSL_04023	RS04681	Hypothetical protein transmembrane protein
	2.56	RRSL_04046		Hypothetical protein
	2.09	RRSL_04065		Hypothetical protein
	-2.23	RRSL_04070		Hypothetical protein
	-2.92	RRSL_04078	RSc0597	Hypothetical protein
	-3.08	RRSL_04079		Hypothetical protein
	-2.33	RRSL_04182	RSc3401	Shikimate kinase (EC:2.7.1.71)
	-2.02	RRSL_04185	RSc3404	Hypothetical protein
	2.25	RRSL_04400	RSc1003	Carboxypeptidase S1 (EC:3.4.16.6)
	2.04	RRSL_04446		Hypothetical protein
	2.37	RRSL_04447		Hypothetical protein
	-2.05	RRSL_04450	RSc0951	Hypothetical cytosolic protein
	-3.41	RRSL_04451	RSc0950	Hypothetical protein
	2.11	RRSL_04471	RSc0930	probable transmembrane protein
	2.39	RRSL_04496	RS03672	Hemolysin-type Calcium-binding protein - RTX toxin
	2.02	RRSL_04500	RS00951	Hypothetical protein
	2.02	RRSL_04505		Hypothetical protein
	2.6	RRSL_04506		Hypothetical protein
	2.02	RRSL_04591		Hypothetical protein
	-2.26	RRSL_04602		Hypothetical protein
	-2.56	RRSL_04675	RS03144	Type I secretion outer membrane protein
	-2.16	RRSL_04678	RS03141	Acriflavin resistance periplasmic protein
	2.1	RRSL_04740	RSp1113	Transmembrane multidrug-efflux system lipoprotein
	-4.35	RRSL_04750	RSp1105	Alpha,alpha-trehalose-phosphate synthase (UDP-forming) (EC:2.4.1.15)
	-3.42	RRSL_04751	RSp1104	Trehalose-phosphatase (EC:3.1.3.12)
	2.09	RRSL_04787	RS02314	Hypothetical protein
	-2.3	RRSL_RNA022		(tRNA)
	-2.2	RRSL_RNA034		(tRNA)

[a]Fold change was calculated based on gene expression at 20°C compared to 28°C in CPG. Positive values indicate up-regulation of genes at 20°C, and negative values indicate down-regulation of genes at 20°C.
[b]The GMI1000 locus tag is shown if strain GMI1000 has a corresponding ortholog.

. *R. solanacearum* strain UW551 genes differentially expressed in rich culture medium (CPG) at 20°C compared to 28°C.

Gene symbol	Fold-change[a]	UW551 locus tag	GMI1000 Locus tag[b]	Gene product
	-2.18	RRSL_00067	RS03728	Peptide synthetase
	-3.04	RRSL_00121	RSp0239	1,4-alpha-glucan branching enzyme (EC:2.4.1.18)
	-2.52	RRSL_00122	RS05183	Trehalose synthase
	2.01	RRSL_00168	RS01956	Hypothetical protein
	2.44	RRSL_00256		Hypothetical protein
	2.01	RRSL_00419		Hypothetical protein
hrcT	2.64	RRSL_00525	RSp0872	HrcT
	2.12	RRSL_00530		Hypothetical protein
	-2.25	RRSL_00545	RSc1789	Hypothetical protein
	3.57	RRSL_00575	RS02288	Hypothetical protein
	-2.02	RRSL_00616	RSp0006	UTP--glucose-1-phosphate uridylyltransferase (EC:2.7.7.9)
	-2.76	RRSL_00744		Porin
	-2.36	RRSL_00745	RS02001	Hypothetical protein
	2.18	RRSL_00754	RSc0741	Probable chemotaxis response regulator protein (cheY2)
	-2.5	RRSL_00833		Hypothetical protein
	3	RRSL_00840		Hypothetical protein
	-2.8	RRSL_00920	RS05439	Molybdopterin-guanine dinucleotide biosynthesis protein A
	-4.1	RRSL_00921	RS05440	Hypothetical protein
mexC	2.34	RRSL_00937	RSp0313	MexC
mexD	2.43	RRSL_00938	RSp0312	MexD
	2.47	RRSL_00939	RSp0311	Type I secretion outer membrane protein
	-2.02	RRSL_00977	RSc2358	Phosphoenolpyruvate carboxylase (EC:4.1.1.31)
	2.93	RRSL_01069		Hypothetical protein
	2.48	RRSL_01070		Hypothetical protein
	2.69	RRSL_01114		Hypothetical protein
	2.59	RRSL_01249		Adenine DNA methyltransferase-like protein
	2.26	RRSL_01250		Hypothetical protein
	3.64	RRSL_01251		Hypothetical protein
	-2.04	RRSL_01301	RSc1737	Outer membrane porin protein 32 precursor
	3.91	RRSL_01308	RSc1730	Short-chain type dehydrogenase/reductase (EC:1.-)
	3.13	RRSL_01309	RSc1729	Transcriptional regulators, LysR family
	3.24	RRSL_01310	RSc1728	Hypothetical protein
	2.4	RRSL_01312	RSc1726	Hypothetical protein
	2.6	RRSL_01313	RSc1725	Hypothetical protein
	-2.06	RRSL_01342		Membrane-fusion protein (Cell envelope biogenesis, outer membrane)
	-2.02	RRSL_01364		Molybdopterin biosynthesis MoeB protein
	-2.72	RRSL_01365		MEC+
	-2.6	RRSL_01366		ThiS family protein
	-2.25	RRSL_01367		Bile acid-inducible operon protein F
	-2.13	RRSL_01368		2,3-dihydroxybenzoate-AMP ligase (EC:2.7.7.58)
	-2.16	RRSL_01369		Acyl-CoA dehydrogenase (EC:1.3.99.-)
	-2.14	RRSL_01372		Hypothetical protein
	-2.12	RRSL_01373		Possible TonB-dependent receptor
	-2.37	RRSL_01387		Hypothetical protein
	2.17	RRSL_01401	RSc1276	Cytochrome c oxidase polypeptide I (EC:1.9.3.1)

	-5.56	RRSL_01417		Hypothetical protein
	-2.05	RRSL_01451	RSc2526	Superoxide dismutase (EC:1.15.1.1)
	-4.91	RRSL_01475	RSc2502	Hypothetical protein
	-2.44	RRSL_01476		Hypothetical protein
	-2.37	RRSL_01539	RSc1335	ClpB protein
glgA	-3.82	RRSL_01693	RSp0242	Probable glycogen synthase, glgA (EC:2.4.1.21)
	-2.2	RRSL_01826	RS05802	Hypothetical protein
	-2.65	RRSL_01827	RS00392	Mechanosensitive ion channel
	2.29	RRSL_02047		Hypothetical protein
	2.02	RRSL_02048		Hypothetical protein
	3.99	RRSL_02063		Hypothetical protein
	2.77	RRSL_02066		Hypothetical protein
ptsA	2.06	RRSL_02178	RSc1531	PstA
	2.98	RRSL_02354	RSc2703	Hypothetical protein
	3.08	RRSL_02355		Bacterial protein Translation Initiation Factor 1 (IF-1)
	5.11	RRSL_02356		Hypothetical protein
	-2.17	RRSL_02512		Putative osmotically inducible lipoprotein B1 transmemberane-related protein
	-2.08	RRSL_02560		Conserved Hypothetical protein
	2.24	RRSL_02625		Hypothetical protein
	-2.36	RRSL_02654	RSc2803	Hypothetical protein
	2.38	RRSL_02785	RSc3292	Integral membrane protein
lecM	7.19	RRSL_02788	RSc3288	Mannose/ fucose-binding lectin
aidA	9.58	RRSL_02789		AidA
aidC	3.55	RRSL_02790		Hypothetical protein
solR	2.4	RRSL_02791	RSc3287	SolR
solI	2.09	RRSL_02792	RSc3286	SolI
	-3.49	RRSL_02891	RSc3083	Hypothetical protein
	-2.6	RRSL_02892		Hypothetical protein
	-3.79	RRSL_02923	RSc3053	Hypothetical protein
	-2.43	RRSL_02955	RSc0023	Hypothetical protein
	-2.16	RRSL_03027	RS00875	Ornithine cyclodeaminase (EC:4.3.1.12)
	-2.47	RRSL_03030	RS00878	Hypothetical IucA protein (EC:6.-)
	-2.51	RRSL_03031	RS00879	Hypothetical IucC protein (EC:6.-)
	-2.05	RRSL_03032	RS00880	4-hydroxy-2-oxovalerate aldolase (EC:4.1.2.)
	-3.15	RRSL_03038	RS02188	Cyanate lyase (EC:4.2.1.104,EC:4.3.99.1)
	2.75	RRSL_03068	RS02205	Hypothetical protein
	-2.21	RRSL_03084	RS02224	Hypothetical protein
	-2.85	RRSL_03085	RS02225	Hypothetical protein
	-2.53	RRSL_03086	RS02226	Two-component response regulator
	-4.35	RRSL_03089	RS02229	Hypothetical protein
rpoN	-2.11	RRSL_03090	RSp1671	RpoN
	-2.27	RRSL_03093	RS02233	Hypothetical protein
	-2.75	RRSL_03094	RS02235	Hypothetical protein
	-2.91	RRSL_03096		Hypothetical protein
nac	-2.38	RRSL_03134	RSp0942	Nac
	-2.38	RRSL_03135	RSp0941	Hypothetical protein
	2.26	RRSL_03161	RS02477	Probable hemagglutinin-related protein
	-3.81	RRSL_03167	RS02580	NAD-dependent oxidoreductase
	-2.69	RRSL_03184	RS02600	putative hemagglutinin-related transmembrane protein
	-2.15	RRSL_03321	RSc0341	Hypothetical protein
	2.07	RRSL_03353		Hypothetical protein

	2.18	RRSL_03366	RS02393	Omega-amino acid--pyruvate aminotransferase (EC:2.6.1.18)
	-2.15	RRSL_03395	RSp0122	Methylisocitrate lyase (EC:4.1.3.30)
	2.68	RRSL_03527	RSc2238	Hypothetical protein
	2.07	RRSL_03700		Hypothetical protein
glgX	-2.21	RRSL_03726	RSp0238	GlgX
	-3.48	RRSL_03776	RSc0443	Hypothetical protein
	-2.15	RRSL_03777	RSc0444	Hypothetical protein
	2.09	RRSL_03828		Hypothetical protein
	-3.1	RRSL_03846	RSc2466	Cold shock protein
	2.17	RRSL_03922		Hypothetical protein
	2.27	RRSL_03923		Hypothetical protein
	2.11	RRSL_03933		(partial?) integrase
	-3.32	RRSL_03978	RSc3128	Aldehyde dehydrogenase (EC:1.2.1.3)
	-2.22	RRSL_04023	RS04681	Hypothetical protein transmembrane protein
	2.56	RRSL_04046		Hypothetical protein
	2.09	RRSL_04065		Hypothetical protein
	-2.23	RRSL_04070		Hypothetical protein
	-2.92	RRSL_04078	RSc0597	Hypothetical protein
	-3.08	RRSL_04079		Hypothetical protein
	-2.33	RRSL_04182	RSc3401	Shikimate kinase (EC:2.7.1.71)
	-2.02	RRSL_04185	RSc3404	Hypothetical protein
	2.25	RRSL_04400	RSc1003	Carboxypeptidase S1 (EC:3.4.16.6)
	2.04	RRSL_04446		Hypothetical protein
	2.37	RRSL_04447		Hypothetical protein
	-2.05	RRSL_04450	RSc0951	Hypothetical cytosolic protein
	-3.41	RRSL_04451	RSc0950	Hypothetical protein
	2.11	RRSL_04471	RSc0930	probable transmembrane protein
	2.39	RRSL_04496	RS03672	Hemolysin-type Calcium-binding protein - RTX toxin
	2.02	RRSL_04500	RS00951	Hypothetical protein
	2.02	RRSL_04505		Hypothetical protein
	2.6	RRSL_04506		Hypothetical protein
	2.02	RRSL_04591		Hypothetical protein
	-2.26	RRSL_04602		Hypothetical protein
	-2.56	RRSL_04675	RS03144	Type I secretion outer membrane protein
	-2.16	RRSL_04678	RS03141	Acriflavin resistance periplasmic protein
	2.1	RRSL_04740	RSp1113	Transmembrane multidrug-efflux system lipoprotein
	-4.35	RRSL_04750	RSp1105	Alpha,alpha-trehalose-phosphate synthase (UDP-forming) (EC:2.4.1.15)
	-3.42	RRSL_04751	RSp1104	Trehalose-phosphatase (EC:3.1.3.12)
	2.09	RRSL_04787	RS02314	Hypothetical protein
	-2.3	RRSL_RNA022		(tRNA)
	-2.2	RRSL_RNA034		(tRNA)

[a]Fold change was calculated based on gene expression at 20°C compared to 28°C in CPG. Positive values indicate up-regulation of genes at 20°C, and negative values indicate down-regulation of genes at 20°C.
[b]The GMI1000 locus tag is shown if strain GMI1000 has a corresponding ortholog.
