# Supplementary material for: Comparative Transcriptome Analysis Reveals Cool Virulence Factors of Ralstonia solanacearum Race 3 Biovar 2
Source: PLoS One. 2015 Oct 7;10(10):e0139090. doi: 10.1371/journal.pone.0139090 (PMC4596706; doi:10.1371/journal.pone.0139090)
Supplement: S4 Table — (PDF) [file pone.0139090.s008.pdf]

S4 Table. *R. solanacearum* strain GMI1000 genes differentially expressed *in planta* at 20°C compared to 28°C.

Gene symbol	Fold-change[a]	GMI1000 locus tag	UW551 locus tag[b]	Gene product
acrB	2.6	RSc0010	RRSL_02941	Putative acriflavin resistance transmembrane protein
acrA	3.78	RSc0011	RRSL_02942	Probable acriflavin resistance lipoprotein a precursor
acrR	2.71	RSc0012	RRSL_02943	Putative acrab operon repressor transcription regulator protein
	-2.46	RSc0030	RRSL_02963	Putative transcription regulator protein
	-2.28	RSc0032	RRSL_02968	Hypothetical protein
mreD	-3.12	RSc0061	RRSL_02018	Probable rod shape-determining mred transmembrane protein
	2.27	RSc0064	RRSL_02015	Putative long-chain-fatty-acid--CoA ligase protein (EC:6.2.1.-)
	2.38	RSc0065	RRSL_02014	Probable transcription regulator protein
smf	-2.45	RSc0068	RRSL_02011	Probable smf protein
	-3.34	RSc0095		Hypothetical protein
	-8.22	RSc0096		Hypothetical protein
	-2.75	RSc0105		Hypothetical protein
	-2.3	RSc0124	RRSL_04347	Hypothetical protein
	-2.46	RSc0125	RRSL_04346	Hypothetical protein
	-2.51	RSc0135	RRSL_04334	Lipid A biosynthesis lauroyl acyltransferase (EC:2.3.1.-)
	-3.01	RSc0143	RRSL_04326	Hypothetical protein
	-2.27	RSc0144	RRSL_04325	Probable transcription regulator protein
	-2.69	RSc0145	RRSL_04324	Putative transmembrane protein
	-2.25	RSc0147[c]	RRSL_04322	Hypothetical signal peptide protein
	-2.39	RSc0153	RRSL_04316	Hypothetical signal peptide protein
	2.83	RSc0215		Short chain dehydrogenase (EC:1.-)
	-2.71	RSc0217	RRSL_02876	Hypothetical oxidoreductase transmembrane protein
	-2.91	RSc0232		Hypothetical protein
	-2.48	RSc0240		Hypothetical protein
	2.97	RSc0297	RRSL_01648	Hypothetical protein
	-3.29	RSc0303		Hypothetical protein
	-2.39	RSc0317	RRSL_01628	Probable transmembrane protein
	3.76	RSc0320	RRSL_01626	Hypothetical protein
nasE	-2.27	RSc0380	RRSL_03281	Putative nitrate transmembrane abc transporter protein
	-4.63	RSc0423		Probable remnant of a transposase protein
	-2.22	RSc0439	RRSL_03772	Putative lipase/esterase protein(EC:3.1.-)
	-3.18	RSc0443	RRSL_03776	Probable glycin-rich signal peptide protein
	2.57	RSc0459	RRSL_03792	Hypothetical protein
	-2.97	RSc0463	RRSL_03796	Probable sodium/solute symporter transmembrane protein
	-2.35	RSc0470	RRSL_03802	Hypothetical protein
	2.51	RSc0472	RRSL_03804	Putative transcription regulator protein
	2.43	RSc0473	RRSL_03805	Putative acyl-coa dehydrogenase oxidoreductase protein(EC:1.3.99.3)
	2.55	RSc0474	RRSL_03806	Putative 3-hydroxyacyl-coa dehydrogenase oxidoreductase protein

	3.68	RSc0475	RRSL_03807	Acetyl-coa acetyltransferase (EC:2.3.1.16)
	2.43	RSc0541	RRSL_04136	Probable pirin-like protein
	-2.68	RSc0544		Hypothetical protein
	-2.53	RSc0570	RRSL_04107	Hypothetical protein
	2.58	RSc0596		Hypothetical protein
	3.14	RSc0603	RRSL_04071	Probable transmembrane protein
avrA	2.91	RSc0608	RRSL_01581	Avra protein
	-2.69	RSc0645		Hypothetical protein
	-2.77	RSc0649	RRSL_00399	Putative transmembrane protein
	-4.23	RSc0716	RRSL_04226	Putative type 4 fimbrial pilin related transmembrane protein
	3.05	RSc0753	RRSL_01498	Probable transmembrane protein
	-2.71	RSc0792		Hypothetical protein
	-3.43	RSc0793	RRSL_02370	Probable transmembrane protein
	5.06	RSc0818	RRSL_02398	Putative endoglucanase protein
	2.48	RSc0819		Hypothetical protein
	-2.68	RSc0838		Hypothetical protein
	-2.66	RSc0849		Hypothetical protein
	-2.43	RSc0854		Hypothetical protein
	-4.06	RSc0858		Probable bacteriophage-related protein
	-2.38	RSc0873		Putative bacteriophage-related transmembrane protein
	-2.51	RSc0875		Putative transmembrane protein
	-4.89	RSc0876		Hypothetical protein
	2.46	RSc0899	RRSL_01087	Probable outer membrane lipoprotein transmembrane
ihfB	3.16	RSc0910	RRSL_00208	Integration host factor beta subunit
	2.25	RSc0931	RRSL_04470	Probable signal peptide protein
	-3.27	RSc0982	RRSL_04419	Probable transmembrane protein
	2.97	RSc1027	RRSL_01193	Probable transmembrane protein
	-2.27	RSc1070	RRSL_01147	Hypothetical protein
	-4.41	RSc1076	RRSL_00913	Probable two-component system response regulator transcription regulator protein
gudD1	-5.43	RSc1079[c]	RRSL_00910	Probable glucarate dehydratase protein(EC:4.2.1.40)
exuT2	-3.73	RSc1080	RRSL_00908	Putative hexuronate transporter transmembrane protein
	2.46	RSc1084	RRSL_00904	Probable porin transmembrane protein
nagL	3.1	RSc1085	RRSL_00903	Putative gst-related protein (EC:2.5.1.18,EC:5.2.1.2)
nagK	5.03	RSc1086		Putative isomerase-decarboxylase homolog protein
nagI	5.06	RSc1087	RRSL_00901	Probable gentisate 1,2-dioxygenase oxidoreductase protein (EC:1.13.11.4)
nagAb	5.7	RSc1088	RRSL_00900	Probable ferredoxin subunit of a ring-hydroxylating dioxygenase oxidoreductase protein (EC:1.-)
nagH	3.89	RSc1089	RRSL_00899	Putative salicylate-5-hydroxylase small oxygenase component oxidoreductase protein (EC:1.-)
nagG	5.06	RSc1090	RRSL_00898	Putative salicylate-5-hydroxylase large oxygenase component oxidoreductase protein (EC:1.-)
nagAa	7.31	RSc1091	RRSL_00897	Probable ferredoxin oxidoreductase oxidoreductase protein (EC:1.-)
	2.33	RSc1113	RRSL_00036	Hypothetical protein
	-4.92	RSc1154	RRSL_01923	Putative reductase oxidoreductase protein (EC:1.1.1.274)
	4.76	RSc1201		Probable transcription regulator protein

	2.77	RSc1202	RRSL_02114	Probable flavoprotein nadh-dependent oxidoreductase (EC:1.-)
	2.45	RSc1210	RRSL_02122	Hypothetical protein
	5.35	RSc1262		Hypothetical protein
	2.51	RSc1273	RRSL_01404	Putative lipoprotein
	-2.58	RSc1284	RRSL_01394	Probable transmembrane protein
emrA	2.87	RSc1293	RRSL_00361	Probable multidrug resistance a (translocase) transmembrane protein
	2.64	RSc1294	RRSL_00359	Putative outer membrane chanel lipoprotein
	3.97	RSc1295	RRSL_00358	Putative transcription regulator protein
	2.43	RSc1303		Hypothetical protein
	2.75	RSc1344	RRSL_01549	Hypothetical protein
cysB1	2.27	RSc1348	RRSL_01553	Putative cys regulon transcriptional activator transcription regulator protein
	-2.45	RSc1356	RRSL_01561	Gala protein 4
pcaH	4.63	RSc1441	RRSL_00190	Probable protocatechuate 3,4-dioxygenase (beta chain) oxidoreductase protein (EC:1.13.11.3)
pcaG	4.63	RSc1442	RRSL_00189	Probable protocatechuate 3,4-dioxygenase (alpha chain) oxidoreductase protein (EC:1.13.11.3)
	-3.05	RSc1460	RRSL_00688	Probable methyl-accepting chemotaxis transducer transmembrane protein
creA	-2.41	RSc1471	RRSL_00700	Putative crea signal peptide protein
	-2.3	RSc1473	RRSL_00702	Putative multidrug resistance-like transmembrane protein
	2.36	RSc1483	RRSL_00427	Hypothetical protein
	2.25	RSc1586[c]	RRSL_04512	Probable lipoprotein
	2.97	RSc1615		Putative transmembrane protein
	-3.14	RSc1687		Hypothetical protein
	-3.23	RSc1691		Probable phage hk97 gp17-related protein
	-3.32	RSc1692		Hypothetical protein
	-2.75	RSc1694		Probable phage hk022 gp18-related protein
	-2.36	RSc1703		Probable bacteriophage-related signal peptide protein (EC:3.4.-)
	-2.69	RSc1705		Hypothetical protein
	-2.57	RSc1732[c]	RRSL_01306	Probable transport system permease transmembrane protein
	-2.99	RSc1743		Hypothetical protein
livH1	-2.53	RSc1751	RRSL_01291	Putative amino acid transmembrane transmembrane protein
	-3.16	RSc1755		Putative transposase protein
	-7.73	RSc1772[c]	RRSL_01610	Probable esterase/lipase protein (EC:3.1.1.-)
	-3.32	RSc1783[c]	RRSL_01618	Hypothetical protein
	-2.73	RSc1792[c]	RRSL_00542	Probable transport system permease transmembrane abc transporter protein
	-3.73	RSc1793[c]	RRSL_00541	Probable permease transmembrane abc transporter protein
	-3.63	RSc1808	RRSL_01074	Probable composite atp-binding transmembrane abc transporter protein
	-2.3	RSc1814		Hypothetical protein
	-2.45	RSc1816		Putative transmembrane protein
	-2.3	RSc1820		Hypothetical protein
	-2.27	RSc1833		Putative signal peptide protein
	-2.81	RSc1892	RRSL_00678	Probable two-component system response regulator transcription regulator protein
	-6.36	RSc1926		Putative phage-related transmembrane protein

	-7.41	RSc1927		Hypothetical protein
	-3.78	RSc1928		Probable tail completion-like protein
	-3.86	RSc1929		Probable tail completion-like protein
	-2.55	RSc1930		Probable signal peptide protein
hemP	2.41	RSc1966	RRSL_01685	Putative hemin uptake protein
	2.23	RSc1971		Hypothetical protein
osmB1	4.59	RSc1973		Putative osmotically inducible lipoprotein b1 transmembrane
	-5.86	RSc2018	RRSL_02463	Probable transcription regulator protein
	2.53	RSc2019	RRSL_02464	Probable signal peptide protein
	2.6	RSc2020	RRSL_02462	Probable acyl-coa dehydrogenase oxidoreductase protein (EC:1.3.99.3)
	-3.84	RSc2028		Hypothetical protein
	-3.94	RSc2039	RRSL_03913	Probable permease transporter transmembrane protein
	-2.43	RSc2040	RRSL_03912	Probable permease transporter transmembrane protein
	-3.16	RSc2083[c]	RRSL_03869	Hypothetical protein
	-2.6	RSc2109	RRSL_03246	Probable transmembrane protein
	-2.58	RSc2122		Hypothetical protein
	2.33	RSc2124	RRSL_01278	Probable lipoprotein transmembrane
	-8.11	RSc2131		Hypothetical protein
	-2.41	RSc2133		Probable transmembrane protein
	2.95	RSc2151[c]	RRSL_01284	Probable signal peptide protein
	-3.36	RSc2174	RRSL_03461	Probable transmembrane abc transporter protein
maf	-2.27	RSc2196	RRSL_03482	Maf-like protein
	4.72	RSc2208	RRSL_03495	Putative pirin-like protein
	2.31	RSc2228		Putative carboxymethylenebutenolidase (dienelactone hydrolase) protein (EC:3.1.1.45)
galU1	2.35	RSc2237	RRSL_03526	Probable utp--glucose-1-phosphate uridylyltransferase protein (EC:2.7.7.9)
pobB	2.39	RSc2242	RRSL_03533	4-hydroxybenzoate 3-monooxygenase (EC:1.14.13.2)
pcaB	3.07	RSc2251	RRSL_03542	3-carboxy-cis,cis-muconate cycloisomerase (EC:5.5.1.2)
pacF	2.27	RSc2252	RRSL_03543	Acetyl-coa acetyltransferase (EC:2.3.1.-,EC:2.3.1.9)
pcaJ	2.48	RSc2253	RRSL_03545	Probable 3-oxoadipate coa-transferase subunit b protein (EC:2.8.3.6)
pcaI	2.87	RSc2254	RRSL_03546	Probable 3-oxoadipate coa-transferase subunit a protein (EC:2.8.3.6)
	3.71	RSc2312		Probable two-component response regulator transcription regulator protein
	2.31	RSc2314		Probable transposase protein
	2.22	RSc2316		Putative drug transport transmembrane protein
	2.45	RSc2317		Hypothetical protein
	-2.79	RSc2360	RRSL_03646	Hypothetical protein
	2.31	RSc2404	RRSL_03607	Hypothetical signal peptide protein
	-2.36	RSc2535		Probable transcription regulator protein
trbD	-2.93	RSc2582		Probable conjugal transfer trbd transmembrane protein
	2.87	RSc2652	RRSL_03205	Probable lipoprotein
	-2.71	RSc2661	RRSL_03215	Putative transcription regulator protein
	-2.81	RSc2672	RRSL_03225	Hypothetical protein
	3.41	RSc2697		Hypothetical protein

	3.58	RSc2698		Hypothetical protein
	3.41	RSc2703	RRSL_02354	Hypothetical protein
	3.53	RSc2704		Hypothetical protein
	-2.43	RSc2710	RRSL_03260	Probable lipoprotein
	2.6	RSc2719	RRSL_00720	Putative cytochrome b-561 transmembrane protein
	-4.26	RSc2727[c]	RRSL_00727_1	Probable multidrug resistance transmembrane protein
	-5.54	RSc2728[c]	RRSL_00728	Hypothetical protein
dgoAb	-2.57	RSc2751		Putative galactonate dehydratase protein (EC:4.2.1.6)
	-2.91	RSc2753		Short chain dehydrogenase (EC:1.-)
	-2.51	RSc2754		Hypothetical protein
araH	-2.3	RSc2756		Probable l-arabinose transmembrane abc transporter protein
	-4.29	RSc2794		Hypothetical protein
	-2.25	RSc2798[c]	RRSL_02659	Hypothetical signal peptide protein
	-2.3	RSc2810	RRSL_02646	Probable transmembrane protein
ftsW	-2.38	RSc2845	RRSL_01991	Probable cell division ftsw transmembrane protein
	2.2	RSc2853	RRSL_01983	Hypothetical protein
	-2.68	RSc2856	RRSL_01979	Putative metabolite transport transmembrane protein
fruA	-2.27	RSc2863	RRSL_01972	Probable pts system, fructose-specific iibc component (eiibc-fru) (fructose-permease iibc component) transmembrane protein (EC:2.7.1.69)
	-3.41	RSc2894[c]	RRSL_02730	Hypothetical protein
	-3.23	RSc2895	RRSL_02731	Hypothetical signal peptide protein
	2.28	RSc2897	RRSL_02733	Hypothetical protein
	4.14	RSc2910		Probable signal peptide protein
	-2.36	RSc2918	RRSL_02753	Putative extracytoplasmic function sigma factor (iron-regulated) transcription regulator protein
	-3.05	RSc2920	RRSL_02755	Putative hydroxamate-type ferrisiderophore receptor signal peptide protein
	-2.6	RSc3028	RRSL_00871	Putative signal peptide protein
	-5.03	RSc3063	RRSL_02912	Probable transmembrane sensor histidine kinase transcription regulator protein (EC:2.7.3.-)
	-8.46	RSc3066		Hypothetical protein
	-2.69	RSc3069	RRSL_02907	Putative transporter transmembrane protein
	-2.83	RSc3074	RRSL_02903	Hypothetical protein
	-2.38	RSc3079[c]	RRSL_02899	Probable composite two component regulatory (sensor histidine kinase and response regulator hybrid) transcription regulator protein (EC:2.7.3.-)
	2.64	RSc3083	RRSL_02891	Hypothetical signal peptide protein
	13.64	RSc3092	RRSL_03155	Putative signal peptide protein
	-2.46	RSc3095		Putative serine-rich protein
	2.68	RSc3104	RRSL_04001	Putative calcium binding signal peptide protein
	-3.51	RSc3136	RRSL_03970	Putative methyl-accepting chemotaxis transmembrane protein
	-3.53	RSc3137[c]	RRSL_03968	Hypothetical protein
	-2.3	RSc3140	RRSL_03965	Putative serine protease protein (EC:3.4.21.-)
	-3.58	RSc3148		Probable transmembrane protein
	2.27	RSc3206		Probable transport lipoprotein
	-2.51	RSc3261	RRSL_02821	Hypothetical protein
	3.51	RSc3267[c]	RRSL_02815	Putative lipoprotein
	-2.57	RSc3271	RRSL_02811	Putative arsenate reductase oxidoreductase protein

dctA3	-2.6	RSc3275[c]	RRSL_02806	C4-dicarboxylate transport protein
	-2.6	RSc3279	RRSL_02801	Hypothetical protein
hyi	-6.41	RSc3282	RRSL_02798	Probable hydroxypyruvate isomerase protein (EC:5.3.1.22)
	-3.43	RSc3308	RRSL_03671	Probable transcription regulator protein
gst2	2.58	RSc3309	RRSL_03672	Probable glutathione s-transferase protein (EC:2.5.1.18)
	2.79	RSc3349	RRSL_00797	Hypothetical protein
	-2.36	RSc3371	RRSL_00850	Putative transmembrane abc transporter protein
	-2.5	RSc3379	RRSL_00846	Putative transmembrane protein
kdpA	-2.97	RSc3382[c]	RRSL_00844	Potassium-transporting atpase subunit A (EC:3.6.1.36,EC:3.6.3.12)
	2.5	RSc3387	RRSL_04164	Probable two-component response regulator transcription regulator protein
	-2.45	RSc3395[c]	RRSL_04174	Putative transmembrane protein
	-3.53	RSc3400	RRSL_04180	Probable tranporter transmembrane protein
	-2.53	RSc3402	RRSL_04183	Probable transmembrane protein
	-2.3	RSc3405	RRSL_04186	Probable transmembrane protein
galU2	6.28	RSp0006	RRSL_00616	Probable utp--glucose-1-phosphate uridylyltransferase udp-glucose pyrophosphorylase protein (EC:2.7.7.9)
	7.84	RS05541		Hypothetical protein
	33.13	RS05540	RRSL_03422	Hypothetical protein
	3.78	RS05556		Hypothetical protein
	4.69	RS03015	RRSL_03409	Putative transport transmembrane protein
egl	2.85	RSp0162	RRSL_04288	Endoglucanase precursor (endo-1,4-beta-glucanase) protein (EC:3.2.1.4)
	2.53	RS05515	RRSL_04287	Putative phosphopantetheinyl transferase protein
	-2.77	RS05513	RRSL_04285	Hypothetical protein
	-2.83	RS04694		Probable transporter transmembrane protein
	-3.2	RS04703		Hypothetical protein
	2.48	RS05216	RRSL_03556	Hypothetical protein
	3.05	RS05215		Hypothetical protein
vanA	6.54	RSp0222	RRSL_03707	Probable vanillate o-demethylase oxygenase subunit oxidoreductase protein (EC:1.14.13.- ,EC:1.14.13.82)
vanB	4.29	RSp0223	RRSL_03708	Probable vanillate o-demethylase (vanillate degradation ferredoxin-like) oxidoreductase protein (EC:1.14.13.-)
fca	5.7	RSp0225	RRSL_03710	Enoyl-coa hydratase
vdh	5.62	RSp0226	RRSL_03711	Probable vanillin dehydrogenase oxidoreductase protein (EC:1.-,EC:1.2.1.65)
fcs	2.87	RSp0227		Feruloyl-coa synthase
	-5.9	RS05192	RRSL_03718	Hypothetical signal peptide protein
	-4.2	RS05191	RRSL_03719	Probable esterase/lipase protein (EC:3.1.-)
	-4.5	RS05189	RRSL_03722	Probable metabolite transport transmembrane protein
	2.55	RS05183	RRSL_00122	Hypothetical protein
	2.27	RS05182	RRSL_01692	Putative alpha-amylase-related protein
glgA	2.62	RSp0242	RRSL_01693	Glycogen synthase (EC:2.4.1.21)
	-3.71	RS03722	RRSL_01698	Probable outer membrane hemin receptor signal peptide protein
	2.55	RS03717[c]	RRSL_01704	Hypothetical protein
cysK1	-2.27	RSp0276		Probable cysteine synthase a protein (EC:4.2.99.8)

	-2.62	RS03673		Probable transmembrane protein
	3.2	RS05453	RRSL_00935	Probable transmembrane sensor kinase transcription regulator protein (EC:2.7.-)
	8.94	RS05452	RRSL_00934	Putative transmembrane protein
	10.85	RS05451	RRSL_00933	Hypothetical protein
	2.38	RS05447	RRSL_00929	Probable transcription regulator protein
	2.3	RS05442	RRSL_00923	Probable dehydrogenase, small chain oxidoreductase protein (EC:1.-)
flgM	3.81	RSp0340	RRSL_02346	Probable negative regulator of flagellin synthesis (anti-sigma-28 factor) protein
	-2.46	RS00792		Acyl carrier protein
	-3.12	RS00794		Putative acyl-carrier-protein
	-2.91	RS00796		Putative 1-acyl-sn-glycerol-3-phosphate acyltransferase alpha transmembrane protein (EC:2.3.1.51)
pat	-2.45	RSp0397		Probable phosphinothricin acetyltransferase protein (EC:2.3.1.-)
	9.06	RS00831	RRSL_02300	Hypothetical signal peptide protein
	2.53	RS00866		Hypothetical protein
fsr	-3.05	RSp0412	RRSL_03020	Putative fosmidomycin resistance antibiotic resistance transmembrane protein
cysK2	2.43	RSp0417	RRSL_03026	Probable cysteine synthase a protein (EC:4.2.99.8)
	2.48	RS00875	RRSL_03027	Probable ornithine cyclodeaminase protein (EC:4.3.1.12)
	2.57	RS00359	RRSL_01795	Probable substrate-binding periplasmic (pbp) abc transporter protein
	2.23	RS00362	RRSL_01798	Hypothetical protein
	-2.41	RS00373		Hypothetical protein
	3.03	RS00392	RRSL_01827	Probable transmembrane protein
	-2.48	RS00415	RRSL_00367	Probable transcription regulator protein
	-3.01	RS00416		Probable sugar-proton symporter transmembrane protein
	-2.6	RS03895		Truncated TIS1421-transposase protein A
	2.5	RS03879		Hypothetical protein
phnB	2.68	RSp0601		Hypothetical protein
	-2.51	RS03758	RRSL_01784	Hypothetical protein
cbiD	-2.28	RSp0622	RRSL_02995	Probable cobalamin biosynthesis d transmembrane protein
	-2.81	RS03732	RRSL_03009	Putative acyltransferase transmembrane protein (EC:2.3.1.-)
	-2.51	RS05577	RRSL_02242	Probable transcription regulator protein
	2.23	RS01741	RRSL_02232	Probable signal peptide protein
	-2.43	RS01957	RRSL_01741	Hypothetical protein
	-2.22	RS01940		Putative vgr-related protein
	-2.55	RS01938		Hypothetical protein
	-2.39	RS01937		Hypothetical protein
	-2.25	RS01936		Hypothetical signal peptide protein
	-2.77	RS01934		Hypothetical protein
	-2.2	RS01918		Hypothetical protein
	-2.93	RS01917		Putative hydrolase protein (EC:3.-)
	9.32	RS01912		Hypothetical protein
	2.22	RS01904		Probable signal peptide protein
	-2.25	RS01899	RRSL_00107	Probable hemagglutinin-related transmembrane protein

	-6.32	RS01896	RRSL_00126	Probable tonb-dependent receptor signal peptide protein
	2.97	RS01888	RRSL_01041	Probable drug efflux lipoprotein
	-3.92	RS05372	RRSL_01037	Probable transcription regulator protein
gudP	-2.36	RSp0828	RRSL_01031	Putative glucarate transporter (d-glucarate permease) transmembrane protein
	-2.87	RS05350		Awr family protein
	-2.45	RS05349		Probable Awr family protein
hrpW	2.58	RSp0857	RRSL_00510	Hrpw transmembrane protein
hrpJ	2.5	RSp0866		Hrpj protein
hrpB	2.2	RSp0873[c]	RRSL_10003	Regulatory hrpb transcription regulator protein
	-2.85	RS01664		4-oxalocrotonate tautomerase (EC:5.3.2.-)
	4.11	RS01668		Probable signal peptide protein
	-2.38	RS01677		Putative signal peptide protein
nac	2.25	RSp0942	RRSL_03134	Probable nitrogen assimilation regulatory transcription regulator protein
nrdG	-2.66	RSp0965	RRSL_04609	Putative ribonucleotide reductase activating transmembrane protein
	-3.51	RS02303		Probable transmembrane protein
narK1	-2.33	RSp0972	RRSL_04607	Putative nitrite/nitrate transporter transmembrane protein
narK2	-2.91	RSp0973	RRSL_04606	Probable nitrite/nitrate transporter transmembrane protein
narG	-2.5	RSp0974	RRSL_04603	Probable respiratory nitrate reductase alpha chain oxidoreductase protein (EC:1.7.99.4)
narJ	-2.91	RSp0976	RRSL_04600	Putative respiratory nitrate reductase oxidoreductase protein (EC:1.7.99.4)
narI	-3.1	RSp0977	RRSL_04599	Probable respiratory nitrate reductase transmembrane protein (EC:1.7.99.4)
mobB1	-2.43	RSp0978	RRSL_04598	Probable molybdopterin-guanine dinucleotide biosynthesis protein
	-4.44	RS02321	RRSL_02360	Probable transmembrane protein
dctA2	-2.85	RSp0995		Probable c4-dicarboxylate transport transmembrane protein
	-2.31	RS02364	RRSL_00493	Putative methyl-accepting chemotaxis transducer transmembrane protein
	-2.85	RS02368		Probable transmembrane protein
	-2.38	RS02369	RRSL_00832	Hypothetical protein
	-2.83	RS02383	RRSL_00819	Probable amino acid transporter transmembrane protein
	-3.76	RS02587	RRSL_03171	Probable transmembrane protein
	2.25	RS02604	RRSL_04758	Putative hns-like transcription regulator protein
otsB	2.71	RSp1104	RRSL_04751	Probable trehalose-phosphatase protein (EC:3.1.3.12)
	5.62	RS02618	RRSL_04743	Probable transcription regulator protein
	3.63	RS02619	RRSL_04742	Probable lipase protein (EC:3.1.1.-)
	5.98	RSp1112	RRSL_04741	Probable transmembrane multidrug efflux system transmembrane protein
	3.12	RSp1113	RRSL_04740	Probable transmembrane multidrug-efflux system lipoprotein transmembrane
	2.51	RS05506	RRSL_04739	Putative outer-membrane drug efflux protein
	2.41	RS05504	RRSL_03755	Putative low specificity l-threonine aldolase protein (EC:4.1.2.5)
	3.1	RS05502		Putative transmembrane protein
	3.68	RS05501		Hypothetical protein

	4.79	RS05500		Hypothetical protein
	2.85	RS05499		Hypothetical protein
	3.34	RS05497		Hypothetical protein
	2.51	RS05494		Putative nadp-dependent zinc-type alcohol dehydrogenase oxidoreductase protein (EC:1.1.1.- ,EC:1.1.1.255)
	3.48	RS05474[c]	RRSL_04734	Probable transcription regulator protein
	-4.23	RS05467[c]	RRSL_04731	Probable transmembrane protein
	-4.96	RS05071	RRSL_04694	Putative outer membrane efflux transmembrane protein
	-2.5	RS05072	RRSL_04693	Putative composite atp-binding transmembrane abc transporter protein
	-3.43	RS03136	RRSL_04683	Probable transcription regulator protein
	-2.55	RS03162		Hypothetical protein
	-2.73	RS03167	RRSL_01120	Probable high affinity nitrate transporter transmembrane protein
aer	-2.83	RSp1224	RRSL_01121	Probable aerotaxis sensor receptor (chemotaxis transducer) transmembrane protein
	-3.56	RS03175	RRSL_01131	Putative transferase protein (EC:2.-)
	-12.64	RS03178	RRSL_01134	Putative transferase protein (EC:2.-)
	-5.98	RS03179	RRSL_01135	Probable transcription regulator protein
	-3.43	RS03190	RRSL_00375	Probable lipoprotein
	-3.34	RS05307	RRSL_00391	Hypothetical protein
	-2.45	RS05313	RRSL_00336	Probable integral membrane transmembrane protein
	-2.6	RS05317		Probable transmembrane protein
	-2.27	RS05322	RRSL_02855	Putative transmembrane protein
	-2.55	RS05325	RRSL_03374	Probable sugar kinase protein (EC:2.7.1.-)
	-5.24	RS05326	RRSL_03375	Hypothetical protein
	-3.41	RS05327	RRSL_03376	Probable multifunctional protein : phosphocarrier protein hpr (protein h) and phosphoenolpyruvate-protein phosphotransferase (EC:2.7.3.9)
	-2.91	RS05328	RRSL_03377	Probable porin transmembrane protein
scrB	-4.89	RSp1284	RRSL_03378	Putative sucrose-6-phosphate hydrolase (sucrase invertase) protein (EC:3.2.1.26)
scrA	-3.78	RSp1285	RRSL_03379	Probable pts system, sucrose-specific (iibc component) transmembrane protein (EC:2.7.1.69)
	2.71	RS05664		Hypothetical protein
	2.22	RS05659		Putative transcription regulator protein
	2.57	RS02091		Probable lipoprotein
nosY	-3.58	RSp1372		Probable nosy transmembrane protein
	-2.6	RS02083		Hypothetical protein
flhA	-2.77	RSp1393	RRSL_00581	Probable flagellar biosynthesis flha transmembrane protein
	-2.77	RS02062	RRSL_00584	Putative transporter transmembrane protein
	2.48	RS03115		Probable transmembrane protein
	2.55	RS03114		Hypothetical protein
	2.99	RS03111		Putative oxidoreductase signal peptide protein (EC:1.-)
	2.41	RS03110		Hypothetical protein
	2.27	RS03109		Hypothetical protein
	2.83	RS03108		Hypothetical protein
	2.28	RS03106		Putative acetyltransferase protein (EC:2.3.1.-)
	3.2	RS03105		Hypothetical protein

	3.12	RS03104		Probable trna synthetase protein (EC:6.1.1.-)
	-6.11	RS06034		Hypothetical protein
ttuD1	-2.28	RSp1449	RRSL_02796	Probable hydroxypyruvate reductase oxidoreductase protein (EC:1.1.1.81)
	-3.2	RS03081		Putative atp-binding abc transporter protein
	2.6	RS03078		Probable signal peptide protein
	-2.22	RS03075		Putative tyrosine specific protein phosphatase
	-2.36	RS03072		Probable 4-carboxymuconolactone decarboxylase protein (EC:4.1.1.44)
	-10.27	RS03065	RRSL_03103	Probable transcription regulator protein
	-6.68	RS03064	RRSL_03102	Hypothetical protein
	2.31	RS03053		Probable transmembrane protein
	-2.33	RS03043	RRSL_04641	Probable signal peptide protein
	-2.79	RS03041	RRSL_04643	Probable signal peptide protein
	-2.2	RS03035	RRSL_04615	Probable transcription regulator protein
	-3.68	RS03033	RRSL_04617	Probable transmembranee abc transporter protein
	-2.62	RS04790	RRSL_04624	Hypothetical protein
	-2.55	RS04800	RRSL_04632	Hypothetical protein
	-2.36	RS04814		Probable transmembrane protein
	2.43	RS02105	RRSL_04790	Hypothetical protein
	3.16	RS02107	RRSL_04788	Hypothetical protein
	-2.27	RS02118	RRSL_04771	Hypothetical protein
	2.68	RS02119	RRSL_04770	Hypothetical protein
	-4.17	RS02136	RRSL_02534	Probable transmembrane protein
	2.71	RS02152	RRSL_02523	Probable metabolite transport transmembrane protein
	3.32	RS02153		Hypothetical protein
	-2.45	RS02171	RRSL_00778	Probable transport transmembrane protein
	2.45	RS02225	RRSL_03085	Probable transmembrane protein
	4.5	RS02228		Hypothetical protein
	7.26	RS02229	RRSL_03089	Hypothetical signal peptide protein
rpoN2	4.96	RSp1671	RRSL_03090	DNA-directed RNA polymerase subunit N
	2.79	RS02231	RRSL_03091	Hypothetical protein
	4.35	RS02233	RRSL_03093	Hypothetical protein
	2.68	RS02235	RRSL_03094	Probable composite two-component regulatory (sensor kinase and response regulator hybrid) transcription regulator protein
tRNA-proL	3.27	RS03937		(tRNA)

[a]Fold change was calculated based on gene expression at 20°C compared to 28°C *in planta.* Positive values indicate up-regulation of genes at 20°C, and negative values indicate down-regulation of genes at 20°C.
[b]The UW551 locus tag is shown if strain UW551 has a corresponding ortholog.
[c]Indicates genes from *R. solanacearum* species complex core genome that were differentially expressed by temperature *in planta* in both strains.
