# Supplementary material for: Comparative Transcriptome Analysis Reveals Cool Virulence Factors of Ralstonia solanacearum Race 3 Biovar 2
Source: PLoS One. 2015 Oct 7;10(10):e0139090. doi: 10.1371/journal.pone.0139090 (PMC4596706; doi:10.1371/journal.pone.0139090)
Supplement: S5 Table — (PDF) [file pone.0139090.s009.pdf]

S5 Table. *R. solanacearum* strain GMI1000 genes differentially expressed in CPG at 20°C compared to 28°C.

Gene symbol	Fold-change[a]	GMI1000 Locus tag	UW551 locus tag[b]	Gene product
	-2.82	RS00416		Probable sugar-proton symporter transmembrane protein
	-2.68	RS00417		Probable transmembrane protein
	-2.11	RS01918		Hypothetical protein
	-2.12	RS01919		Putative transferase protein (EC:2.-)
	-2.16	RS01920		Putative transmembrane protein
	-2.16	RS01941	RRSL_01757	Probable transmembrane protein
	-2.08	RS01959	RRSL_01739	Putative ClpA/B-type chaperone protein
	-2.5	RS01960	RRSL_01738	Hypothetical protein
	-2.11	RS01961	RRSL_01737	Hypothetical protein
	3.19	RS02031		Putative dipeptidase protein (EC:3.4.13.19)
	4.2	RS02032		Hypothetical protein
	2.48	RS02038		Probable ferredoxin oxidoreductase protein
	2.21	RS02043		Probable transcriptional regulator transcription regulator protein
	2.26	RS02287		Hypothetical protein
	2.34	RS02288	RRSL_00575	Hypothetical protein
	2.17	RS02359	RRSL_01066	Hypothetical protein
	-2.07	RS02377		Putative cation efflux system transmembrane protein
	-2.15	RS02378		Probable cation transporter transmembrane protein
	2.01	RS03027		Hypothetical protein
tRNA-serW	-2	RS03460		(tRNA)
	-2.04	RS03737		Probable phospholipase protein
	-2.63	RS03738		Putative vgr-related protein
	2.17	RS03877		Probable signal peptide protein
	3.15	RS03887		Hypothetical protein
	2.87	RS03888		Hypothetical protein
	2.23	RS03910		Hypothetical protein
	2.12	RS03936		Hypothetical protein
	2.07	RS04683	RRSL_04024	Hypothetical protein
	-2.34	RS04687	RRSL_01758	Putative VGR-related protein
	-2.14	RS04689		Hypothetical protein
	-2.09	RS04690	RRSL_04030	Hypothetical protein
	2.1	RS04806	RRSL_04638	Probable 1-aminocyclopropane-1-carboxylate oxidase (acc oxidase) oxidoreductase protein (EC:1.-)
	2.59	RS05218		Hypothetical protein
	2	RS05343	RRSL_02843	Hypothetical protein
	2.12	RS05509		Hypothetical protein
	2.37	RS05526		Putative RHS-related protein
	2.03	RS06029		Hypothetical protein
goaG	-3.36	RSc0029	RRSL_02962	Probable 4-aminobutyrate aminotransferase protein (EC:2.6.1.19)
	-2.17	RSc0158	RRSL_04312	Probable arginase protein (EC:3.5.3.1)
	-3.02	RSc0159	RRSL_04311	Probable acetylornithine aminotransferase protein (EC:2.6.1.13)
avrA	2.04	RSc0608	RRSL_01581	AvrA protein
	2	RSc0617	RRSL_03065	Probable signal peptide protein
	-2.01	RSc0664	RRSL_00414	Probable lipoprotein
	2.07	RSc0824		Hypothetical protein

	2.33	RSc0834		Hypothetical protein
	2.23	RSc0838		Hypothetical protein
	2.42	RSc0851		Hypothetical protein
	2.03	RSc0894		Probable signal peptide protein
	2.59	RSc0952	RRSL_04449	Putative atp-dependent rna helicase protein (EC:3.-)
	-2.35	RSc1076	RRSL_00913	Probable two-component system response regulator transcription regulator protein
sbp	-2.32	RSc1336	RRSL_01540	Probable sulfate-binding precursor signal peptide protein
ssuC	-2.25	RSc1340	RRSL_01545	Putative aliphatic sulfonates transmembrane ABC transporter protein
cysW	-2.06	RSc1346	RRSL_01551	Putative sulfate transport abc transporter protein
	-2.59	RSc1470	RRSL_00699	Hypothetical protein
ppk	-2.01	RSc1536	RRSL_02173	Polyphosphate kinase (EC:2.7.4.1)
	-2.05	RSc1572	RRSL_02596	3-hydroxybutyrate dehydrogenase (EC:1.1.1.30)
	-2.06	RSc1707		Putative transmembrane protein
	-2.14	RSc1796		Putative n-acetylmuramoyl-l-alanine amidase protein (EC:3.5.1.28)
	-2.4	RSc1926		Putative phage-related transmembrane protein
	-2.76	RSc1927		Hypothetical protein
	-2.12	RSc1928		Probable tail completion-like protein
	-2.39	RSc1929		Probable tail completion-like protein
	2.29	RSc2068		Putative signal peptide protein
	3.56	RSc2238	RRSL_03527	Probable transmembrane protein
	2	RSc2312		Probable two-component response regulator transcription regulator protein
	2.52	RSc2313		Putative transposase protein
	2.13	RSc2315		Hypothetical protein
	2.35	RSc2316		Putative drug transport transmembrane protein
	2.55	RSc2317		Hypothetical protein
	4.09	RSc2697		Hypothetical protein
	4.59	RSc2698		Hypothetical protein
	4.76	RSc2699		Hypothetical protein
	3	RSc2700		Hypothetical protein
	3.52	RSc2701		Hypothetical protein
	9.26	RSc2703	RRSL_02354	Hypothetical protein
	4.22	RSc2704		Hypothetical protein
	2.06	RSc2705		Hypothetical protein
	-2.2	RSc3151	RRSL_03953	Putative tryptophan 2-monooxygenase oxidoreductase protein (EC:1.13.12.3)
	2	RSc3191	RRSL_0644_1	Hypothetical protein
	2.05	RSc3214		Hypothetical protein
fdhA	2.17	RSp0053		Probable glutathione-independent formaldehyde dehydrogenase oxidoreductase protein (EC:1.2.1.46)
glyA2	2.51	RSp0055		Serine hydroxymethyltransferase (EC:2.1.2.1)
stcD	2.92	RSp0058		Probable stachydrine utilization oxidoreductase protein
opuC	3.25	RSp0064		Putative glycine betaine transmembrane and periplasmic ABC transporter protein
opuA	2.19	RSp0066		Putative glycine betaine/l-proline atp-binding ABC transporter protein
fliL	2.04	RSp0379	RRSL_02324	Flagellar basal body protein
hrpY	2.1	RSp0855	RRSL_00508	Hrp pilus subunit HrpY protein
popA	2.68	RSp0877	RRSL_02443	PopA protein
katE	-5.21	RSp1581	RRSL_02532	Probable catalase hydroperoxidase HPII oxidoreductase protein (EC:1.11.1.6)

[a]Fold change was calculated based on gene expression at 20°C compared to 28°C in CPG. Positive values indicate up-regulation of genes at 20°C, and negative values indicate down-regulation of genes at 20°C.
[b]The UW551 locus tag is shown if strain UW551 has a corresponding ortholog.
