# Supplementary material for: Comparative Transcriptome Analysis Reveals Cool Virulence Factors of Ralstonia solanacearum Race 3 Biovar 2
Source: PLoS One. 2015 Oct 7;10(10):e0139090. doi: 10.1371/journal.pone.0139090 (PMC4596706; doi:10.1371/journal.pone.0139090)
Supplement: S6 Table — (PDF) [file pone.0139090.s010.pdf]

S6 Table. Genes involved in the salicylic acid degradation pathway were selectively up-regulated in *R. solanacearum* strain GMI1000 during tomato pathogenesis at 20°C.

Gene name	GMI1000 fold change[a]	UW551 fold change[a]	Gene product	GMI1000 locus tag
nagL	3.10	-1.09	Putative glutathione-S-transferase –related (EC:2.5.1.18,EC:5.2.1.2)	RSc1085
nagK	5.03	*n/p*[b]	Putative isomerase-decarboxylase homolog	RSc1086
nagI	5.06	-1.20	Probable gentisate 1,2-dioxygenase oxidoreductase protein (EC:1.13.11.4)	RSc1087
nagAb	5.70	-1.04	Probable ferredoxin subunit of a ring-hydroxyl-ating dioxygenase oxidoreductase (EC:1.-)	RSc1088
nagH	3.89	-1.23	Putative salicylate-5-hydroxylase small oxygenase component oxidoreductase (EC:1.-)	RSc1089
nagG	5.06	-1.22	Putative salicylate-5-hydroxylase large oxygenase component oxidoreductase (EC:1.-)	RSc1090
nagAa	7.31	-1.02	Probable ferredoxin oxidoreductase oxidoreductase (EC:1.-)	RSc1091

[a]Fold change of each gene's expression at 20°C *in planta* compared to 28°C *in planta*. Positive values indicate up-regulation of genes at 20°C, and negative values indicate down-regulation of genes at 20°C.
[b]*n/p* indicates the gene is not present in strain UW551.
