# Supplementary material for: Comparative Transcriptome Analysis Reveals Cool Virulence Factors of Ralstonia solanacearum Race 3 Biovar 2
Source: PLoS One. 2015 Oct 7;10(10):e0139090. doi: 10.1371/journal.pone.0139090 (PMC4596706; doi:10.1371/journal.pone.0139090)
Supplement: S7 Table — (PDF) [file pone.0139090.s011.pdf]

S7 Table. Genes known or putatively involved in multidrug efflux were up-regulated in *R. solanacearum* strain GMI1000 during tomato pathogenesis at 20°C.

Gene name	GMI1000 fold change[a]	UW551 fold change[a]	Gene product	GMI1000 locus tag
emrA	2.87	-2.16	Probable multidrug resistance protein A (EmrA) MLYD family	RSc1293
*n/a**	2.96	-1.12	Probable drug efflux lipoprotein	RS01888
acrB	2.59	1.35	Acriflavin resistance protein (AcrB)	RSc0010
acrA	3.79	2.04	Probable acriflavin resistance lipoprotein A precursor	RSc0011
acrR	2.72	1.26	Repressor of the *acrAB* operon	RSc0012
na[b]	2.51	-1.11	Probable outer membrane drug efflux protein	RS05506
na[b]	3.11	1.05	Transmembrane multidrug-efflux system lipoprotein	RSp1113
na[b]	6.00	1.02	Probable transmembrane multidrug efflux system protein	RSp1112

[a]Fold change of each gene's expression at 20°C *in planta* compared to 28°C *in planta*. Positive values indicate up-regulation of genes at 20°C, and negative values indicate down-regulation of genes at 20°C.
[b] *na* indicates no gene name was available.
