# Supplementary material for: Comparative Transcriptome Analysis Reveals Cool Virulence Factors of Ralstonia solanacearum Race 3 Biovar 2
Source: PLoS One. 2015 Oct 7;10(10):e0139090. doi: 10.1371/journal.pone.0139090 (PMC4596706; doi:10.1371/journal.pone.0139090)
Supplement: S8 Table — (PDF) [file pone.0139090.s012.pdf]

**S8 Table.** Bacterial strains used in this study.

| Strains | Relevant characteristics[a] | Reference or source |
|---|---|---|
| *Escherichia coli* | | |
| DH5α | F- *endA1 relA* ϕ80 *lacZΔM15 hsdR17 supE44 thi-1 recA1 gyrA96* | (Hanahan, 1983) |
| *Ralstonia solanacearum* | | |
| GMI1000 | wild type, Phylotype I seq. 18, isolated from tomato in French Guyana | (Boucher *et al.*, 1985) |
| UW551 | wild type, Phylotype II seq.1, R3bv2 strain isolated from geranium in Wisconsin, U.S.A | (Williamson *et al.*, 2002) |
| UW553 | wild type, Phylotype II seq.1, R3bv2 strain isolated from potato in Guatemala | Allen lab collection |
| UW560 | wild type, Phylotype II seq.1, R3bv2 strain isolated from potato in Guatemala | Allen lab collection |
| GMI1000Rif | Spontaneous rifampicin-resistant variant of GMI1000, fully virulent | (Milling *et al.*, 2009) |
| GMI1000+4149 | GMI1000 carrying the 4,149 bp lecM-aidA-aidC-solR-solI region in the *att* site , $Gm^r$ | This study |
| GMI1000+2354 | GMI1000 carrying the 2,354 bp lecM-aidA-aidC region in the *att* site , $Gm^r$ | This study |
| UW551Rif | Spontaneous rifampicin-resistant variant of UW551, fully virulent | (Swanson *et al.*, 2005) |
| UW551ΔlecM | *lecM* deletion mutant of UW551, $Km^r$ | This study |
| UW551ΔaidA | *aidA* deletion mutant of UW551, $Km^r$ | This study |
| UW551ΔaidC | *aidC* deletion mutant of UW551, $Km^r$ | This study |
| UW551ΔsolI | *solI* deletion mutant of UW551, $Km^r$ | This study |
| UW551RifΔlecM | *lecM* deletion mutant of UW551Rif, $Km^{r,}$ $Rif^r$ | This study |
| UW551 Rif ΔaidC | *aidC* deletion mutant of UW551Rif, $Km^{r,}$ $Rif^r$ | This study |
| UW553ΔlecM | *lecM* deletion mutant of UW553, $Km^r$ | This study |
| UW553ΔaidA | *aidA* deletion mutant of UW553, $Km^r$ | This study |
| UW553ΔaidC | *aidC* deletion mutant of UW553, $Km^r$ | This study |
| UW560ΔlecM | *lecM* deletion mutant of UW560, $Km^r$ | This study |
| UW560ΔaidA | *aidA* deletion mutant of UW560, $Km^r$ | This study |
| UW560ΔaidC | *aidC* deletion mutant of UW560, $Km^r$ | This study |

[a]$Gm^r$, gentamycin resistance; $Km^r$, kanamycin resistance; $Rif^r$, rifampin resistance.

## S8 Table References

Boucher, C., P. Barberis, A. Trigalet & D. Demery, (1985) Transposon mutagenesis of *Pseudomonas solanacearum*: isolation of Tn5-induced avirulent mutants. *J. Gen. Microbiol.* **131**: 2449-2457.

Hanahan, D., (1983) Studies on transformation of *Escherichia coli* with plasmids. *J. Mol. Biol.* **166**: 557-580.

Milling, A., F. Meng, T. P. Denny & C. Allen, (2009) Interactions with hosts at cool temperatures, not cold tolerance, explain the unique epidemiology of *Ralstonia solanacearum* Race 3 biovar 2. *Phytopathology* **99**: 1127-1134.

Swanson, J., J. Yao, J. Tans-Kersten & C. Allen, (2005) Behavior of *Ralstonia solanacearum* Race 3 biovar 2 during latent and active infection of geranium. *Phytopathology* **95**: 136-146.

Williamson, L., N. Kazuhiro, B. Hudelson & C. Allen, (2002) *Ralstonia solanacearum* Race 3, Biovar 2 strains isolated from geranium are pathogenic on potato. *Plant Disease* **86**: 987-991.
