# Supplementary material for: Comparative Transcriptome Analysis Reveals Cool Virulence Factors of Ralstonia solanacearum Race 3 Biovar 2
Source: PLoS One. 2015 Oct 7;10(10):e0139090. doi: 10.1371/journal.pone.0139090 (PMC4596706; doi:10.1371/journal.pone.0139090)
Supplement: S9 Table — (PDF) [file pone.0139090.s013.pdf]

S9 Table. Sequences of PCR primers used in this study.

Primer name	Primer sequences (5'->3')[a]	Product size (bp)	Product /Purpose
Km_F	TCACTATAGGGCGAATTGGG	1,560	Kanamycyn resistance cassette
Km_R	GTTGTGTGGAATTGTGAGCG		
Up lecM_F	CGACGAGAAGATCGAGGAAG	757	*lecM* up stream region
Up lecM_R	cccaattcgccctatagtga AAAGTCGCGTTGTCTGCAC		
Dn lecM_F	cgctcacaattccacacaac GGTTTCCTTTGTCTGTCGG	684	*lecM* down stream region
Dn lecM_R	ACCAAGCCTCGTGACAGC		
Up aidA_F	TCGCCATAACGAACATTCAA	744	*aidA* up stream region
Up aidA_R	cccaattcgccctatagtga TCCGACAGACAAAGGAAACC		
Dn aidA_F	cgctcacaattccacacaac GTGCTGTCACGAGGCTTG	652	*aidA* down stream region
Dn aidA_R	CCACCGGATTGATCTAGTGTC		
Up aidC_F	GGTTTCCTTTGTCTGTCGGA	684	*aidC* up stream region
Up aidC_R	cccaattcgccctatagtga ACCAAGCCTCGTGACAGC		
Dn aidC_F	cgctcacaattccacacaac GGCGGACACTAGATCAATCC	795	*aidC* down stream region
Dn aidC_R	TCTCGATACGGAGCGATACC		
Up solI_F	CTCGTCGCGGATGGACACGG	682	*solI* up stream region
Up solI_R	cccaattcgccctatagtga TACAACGCACTGGCCGGAGG		
Dn solI_F	cgctcacaattccacacaac TGGAAGGGGGCTCGTCCAGG	622	*solI* down stream region
Dn solI_R	AGTGCTCTTCGGCGTGAGCG		
ComWhole_F	CGACGAGAAGATCGAGGAAG	4,149	*lecM* –*solI*

			complementation
ComWhole_R	AACTACATCCAGGGTGCGTC		
Up lecM_F	CGACGAGAAGATCGAGGAAG	2, 534	*lecM-aidC* complementation
Com_aidC_R	GGACGGGCGAAGGCAAGACC		
LecM-F	GGAGACGCACTCTCTATGGC	325	*lecM* gene
LecM -R	CGTTGTAGTCGTTGTCGGTG		
AidA-F	GTTTCCATGTCCCGAATCAC	505	*aidA* gene
AidA-R	CGTTGCTGATGCTGATGAAC		
AidC-F	AAGATGTCCACTCCGCAATC	420	*aidC* gene
AidC-R	GAAGGCATCGGTCTGATGAT		
SolI-F	GCACTATCGGCATCAGGTCT	487	*solI* gene
SolI-R	GATATCGATCCAGCACGCC		
qLecM-F	CGTCGGACCTCGTATCGAA	60	*lecM* qPCR
qLecM -R	CCACCATGGCGAAGTTCAG		
qAidA-F	TGTCATGGTCAACGTCGATACC	65	*aidA* qPCR
qAidA-R	TGGCCATGCTGTTGTTCTTG		
qAidC-F	ATCGTCTTCCTATCCCGATACG	55	*aidC* qPCR
qAidC-R	AGCGTCACGGCATCGAA		
qSolI-F	CCCTATCTGCTGAAGGAAGTCTTT	72	*solI* qPCR
qSolI-R	CACCTCCGGCGATTCG		
qSolR-F	AAGGCAAGACCGCCTACGA	55	*solR* qPCR
qSolR-R	ACCGTGCGCTCGGAGAT		
qCSP-F	TTCTCGGCGATCCAGATGA	52	RRSL_03846 qPCR
qCSP-R	CGCGCTGGCCTTCCT		
qOxyR-F	GGGCAACGGCCACTGTT	55	qPCR control
qOxyR-R	TGAGAGCTCCGGACAGACATT		
qRplM -F	CCGCAAAGCCCCATGAG	54	qPCR control

qRplM -R	TGTCCGTCGCGTCAATCA		
qSerC -F	GGATGACGCGGCTTACGT	54	qPCR control
qSerC -R	TCAACGCCGACGATGGT		

[a]Nucleotide bases in lowercase were added to facilitate SOE-PCR (splicing by overlap extension).
